# Association between use of sodium-glucose cotransporter 2 inhibitors, glucagon-like peptide 1 agonists, and dipeptidyl peptidase 4 inhibitors with kidney outcomes in patients with type 2 diabetes: A systematic review and network meta-analysis

**DOI:** 10.1371/journal.pone.0267025

**Published:** 2022-04-14

**Authors:** Shuo Yang, Wei He, Lu Zhao, Yaochuan Mi

**Affiliations:** Department of Endocrinology and Metabolism, The People’s Hospital of China Medical University, The People’s Hospital of Liaoning Province, Shenyang, Liaoning Province, P.R.China; Universidade Estadual Paulista Julio de Mesquita Filho, BRAZIL

## Abstract

**Background:**

This study aimed to compare the efficacies of sodium-glucose cotransporter 2 (SGLT-2) inhibitors, glucagon-like peptide 1 (GLP-1) agonists, and dipeptidyl peptidase 4 (DPP-4) inhibitors on kidney outcomes in patients with type 2 diabetes using network meta-analysis.

**Methods:**

PubMed, EMBASE, and CENTRAL were searched for studies published up to September 28, 2020. Randomized clinical trials enrolling participants with type 2 diabetes were included, for which SGLT-2 inhibitors, GLP-1 agonists, and DPP-4 inhibitors were compared with either each other, or placebo or no treatment. A network meta-analysis using a Bayesian approach was performed. The primary outcome was composite renal events, and the secondary outcome was acute kidney injury (AKI) events. All research was conducted according to a protocol registered in the PROSPERO database (CRD42020208090).

**Results:**

In total, we retrieved 17 445 studies, of which 98 articles enrolling 186 335 participants were included for the network meta-analysis. For our primary outcome, the network meta-analysis revealed no significant difference between drug classes regardless of baseline factors. However, GLP-1 receptor agonists were most likely ranked best among the three drugs in reducing composite renal events (80%, moderate-quality evidence). Compared with the control groups (OR 0.74, 95% CI 0.62 to 0.87, low-quality evidence), GLP-1 receptor agonists (OR 0.76, 95% CI 0.59 to 0.96, moderate-quality evidence) and with DPP-4 inhibitors (OR 0.67, 95% CI 0.50 to 0.86, low-quality evidence), SGLT-2 inhibitors were associated with a lower risk of AKI events.

**Conclusions:**

In this network meta-analysis, although none of the three new antidiabetic drug classes reduced the composite renal events in participants with type 2 diabetes, GLP-1 receptor agonists may be more effective. The use of SGLT-2 inhibitors was associated with a lower AKI event risk than DPP-4 inhibitors, GLP-1 agonists, placebo, or no treatment.

## Introduction

Diabetic kidney disease (DKD) affects up to 40% of people with diabetes [1], and is an independent risk factor for cardiovascular disease, hypertension, retinal disease, and premature death. Moreover, diabetes accounts for 45% of people with incident end-stage kidney disease [2]. Findings from large randomized controlled trials have shown that chronic renal outcomes can be reduced by intensive glucose control [3], blood pressure lowering, and renin-angiotensin-aldosterone system (RAAS) blockade with either angiotensin-converting enzyme (ACE) inhibitors or angiotensin II receptor blockers (ARBs) [4]. However, even with recommended RAAS blockers, many DKD patients remain at substantial risk for cardio-renal morbidity and mortality [5]. To this end, several novel therapies preventing the progression of DKD have been tested in recent years. There is growing evidence that the use of three newer classes of antihyperglycaemic drugs—sodium-glucose cotransporter 2 (SGLT-2) inhibitors, glucagon-like peptide 1 (GLP-1) agonists, and dipeptidyl peptidase 4 (DPP-4) inhibitors—may reduce the predefined composite renal outcomes [6, 7]. However, most of the current studies have included only patients with established or at risk of cardiovascular disease, limiting its generalizability to other patient populations without these risks.

The onset of acute kidney injury (AKI) was significantly associated with primary adverse outcomes and mortality in patients with diabetes [8]. In 2016, the US Food and Drug Administration (FDA) strengthened the warning on the increased risk of AKI associated with SGLT2 inhibitors [9]. However, growing evidence shows that SGLT-2 inhibitors would decrease the risk of AKI among patients with type 2 diabetes [10]. Better understanding of the risk of AKI among these three classes of glucose-lowering drugs is critically important and will help clinicians decide clinical practice. Little is known about the comparative effects of these three hypoglycemic agents on the risk of AKI. A better understanding of their relationship to AKI risk will help us make clinical practice decisions.

In the last decade, network meta-analysis has become increasingly popular as it synthesizes direct and indirect evidence to compare multiple interventions in a network of randomized controlled trials (RCTs), overcoming the limitations of traditional pare-wise meta-analysis. Considering that the impact of these three classes of hypoglycemic drugs on kidney outcomes remains uncertain, and there are no head-to-head trials among these three agents. Therefore, we conducted comprehensive pairwise and network meta-analyses based on a Bayesian inferential framework to synthesize both direct and indirect evidence from all available randomized controlled trials (RCTs) to compare the efficacy of SGLT-2 inhibitors, GLP-1 agonists, and DPP-4 inhibitors in reducing composite kidney outcomes and AKI risk in participants with type 2 diabetes.

## Methods

This network meta-analysis has been reported following the Preferred Reporting Items for Systematic Reviews and Meta-Analyses (PRISMA-NMA) [11]. All research was conducted according to a protocol registered in the PROSPERO database (CRD42020208090).

### Search strategy

We comprehensively searched PubMed, Embase, and the Cochrane Central Register of Controlled Trials (CENTRAL) from database inception to September 28, 2020. Details of the search strategy are presented in **Appendix 1 in S1 Text**. An additional manual search of the reference lists of included studies, published meta-analyses, and ClinicalTrials.gov was carried out to identify additional trials.

### Study selection

We selected the trials based on the following inclusion criteria: (1) RCTs (either single-blind, double-blind, triple-blind, quadruple-blind, or open-label) in patients with type 2 diabetes mellitus and were published in the English language; (2) Compared SGLT-2 inhibitors, GLP-1 agonists, and DPP-4 inhibitors at market-approved doses with each other or with a control group (defined as placebo or no treatment) (**Appendix 12 in S1 Text**); (3) Trial durations ≥12 weeks; and (4) Trials provided data on any of the predefined primary and secondary outcomes.

Exclusion criteria included a history of diabetic ketoacidosis, type 1 diabetes mellitus (T1DM), pancreas or beta cell transplantation, diabetes caused by pancreatitis, or pancreatectomy.

After removing duplicate records, the author (S.Y.) was responsible for reading the title and abstract of identified studies to assess eligibility. Two authors (W.H. and Y.C.M.) independently extracted data using a standardised data extraction form (**Appendix 13 in S1 Text**). They assessed the full texts of there maining results based on the prespecified inclusion criteria to determine the final included studies. In cases of disagreement, a third author (L.Z.) was consulted to reach a consensus.

### Data extraction and quality assessment

Two reviewers (W.H. and L.Z.) independently extracted data using a predefined data extraction form, based on relevant templates in the Cochrane Handbook for Systematic Reviews [12]. Disagreements were resolved by discussion or consensus with a third reviewer (S.Y.). Data extracted included study characteristics (first author, publication year, study drug and control treatments, and duration of follow-up) and patient characteristics (background treatments, mean age, the proportion of men, duration of T2DM, baseline HbA1c, body mass index, mean eGFR, pre-existing chronic kidney disease (%), pre-existing cardiovascular disease (%) and background ACEi/ARB treatment (%)). If multiple reports from the same population were retrieved, only the most complete and/or most recently reported data would be used. If kidney events were not reported in the manuscripts, data was extracted from the “serious adverse events” section of the Clintrials.gov website. Only data from the randomized controlled periods was used for trials with open-label extension periods. Studies with multiple treatment groups were incorporated into the network as multi-arm studies where appropriate, allowing for multiple pairwise comparisons from a single study to be incorporated into the network without duplication of data. For studies with insufficient information, the reviewers contacted the primary authors, when possible, to acquire and verify the data. We contacted 17 authors of relevant articles, of whom five replied, but they could not provide any additional study data.

The quality of eligible studies was assessed by two authors (Y.C.M. and L.Z.) independently using the Cochrane Collaboration risk of bias tool across five domains (sequence generation, allocation concealment, blinding, detection bias, and attrition bias) [12]. Any disagreements were resolved through consensus. Each domain was judged as low risk, high risk, or unclear risk. Since it was helpful to evaluate the overall quality of evidence from network meta-analysis, the contribution plot was used to analyze the contribution of each direct comparison to the assessment of each network meta-analysis summary effect. Furthermore, a comparison-adjusted funnel plot was used to identify small study effects. Furthermore, the Grading of Recommendations Assessment, Development and Evaluation (GRADE) framework was used to assess the quality of evidence contributing to each network estimate, which characterizes the quality of a body of evidence for the outcomes based on study limitations, imprecision, inconsistency, indirectness, and publication bias [13].

### Outcomes

The primary outcome was composite renal events that included the development of new-onset macroalbuminuria, a decline in estimated glomerular filtration rate (or increase in creatinine), renal impairment, renal failure, and progression of end-stage kidney disease, renal replacement therapy, or renal death [14]. The secondary outcome was acute kidney injury (AKI) events. AKI was defined by each trial or, according to the Medical Dictionary for Regulatory Activities preferred terms for serious adverse events.

### Data synthesis and statistical analysis

The network meta-analysis uses a Bayesian approach and allows comparisons among all studies’ treatment arms, including direct and indirect comparisons simultaneously. A direct meta-analysis was carried out using the DerSimonian and Laird random-effects models to estimate pooled odds ratios and 95% confidence intervals for direct comparisons between therapeutic regimens. Statistical heterogeneity was assessed with the *I* ^2^ statistic, with values over 50% indicating substantial heterogeneity [15]. Analyses were performed using Markov chain Monte Carlo methods in the network meta-analysis. We performed a Bayesian hierarchical network meta-analysis. Fixed-or random-effects models were selected for each outcome based on the deviance information criterion (DIC), using the model with the smallest value (**Appendix 5 in S1 Text**). The transitivity assumption underlying network meta-analysis was evaluated by comparing the distribution of clinical variables that could act as effect modifiers across treatment comparisons [16]. A design-by-treatment test [17] was used to check the assumption of consistency across the entire analytical network. The node splitting method was used to assess the local inconsistency of the model by separating evidence for a particular comparison into direct and indirect evidence. If P > 0.1 in the node-splitting analysis, there would be no significant inconsistency. The global heterogeneity was assessed with the *I*^2^-statistic. The surface under the cumulative ranking curve (SUCRA) and mean ranks were used to rank the treatments for each outcome, which were also built to increase the estimate precision of the relative effect sizes of comparisons, enabling conclusions to be drawn for each outcome of interest.

We compared treatment effects in the following subgroups: (1) Trials with event-driven cardiovascular outcome versus non-cardiovascular outcome; (2) Trials with chronic kidney disease (CKD) versus non-CKD (CKD trial was defined as the mean eGFR <90 ml/min/1.73 m^2^ or the proportion of patients with CKD>60%); (3) Trials with cardiovascular disease (CVD) versus non-CVD (CVD trial was defined as the proportion of patients with CVD>50%); and (4) Trials with ACEi/ARB treatment versus non-ACEi/ARB treatment (ACEi/ARB treatment trial was defined as the proportion of background therapy ACEi/ARB >70%). The sensitivity analysis that was performed was restricted to trial duration (excluding trials with ≤24 weeks of follow-up) and risk of bias (excluding open-label trials). We also performed network metaregressions on BMI at baseline, age, HbA1c, duration of diabetes, and eGFR on the outcomes to better understand the influence of patient characteristics on drug efficacy.

The GeMTC package on R (version 3.6.3) will be used for statistical analysis on network meta-analysis, assessment of global heterogeneity, local inconsistency, and network meta-regression; STATA 14.0 software will be used for pairwise meta-analysis, estimation of global inconsistency, transitivity, and funnel plot.

## Results

### Study search and study characteristics

**Fig 1:**
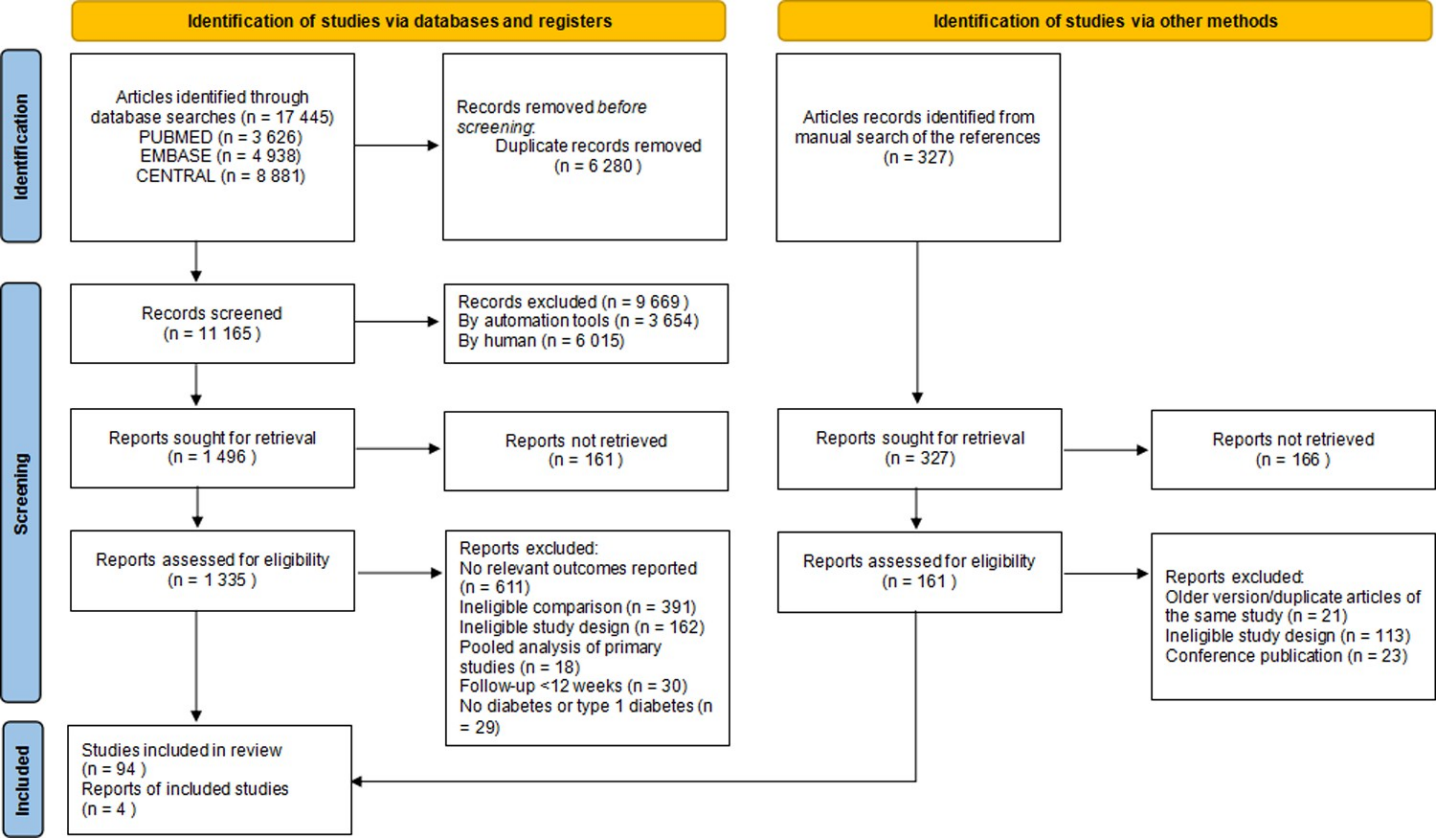
Summary of study retrieval and identification for network meta-analysis.

**Fig 2:**
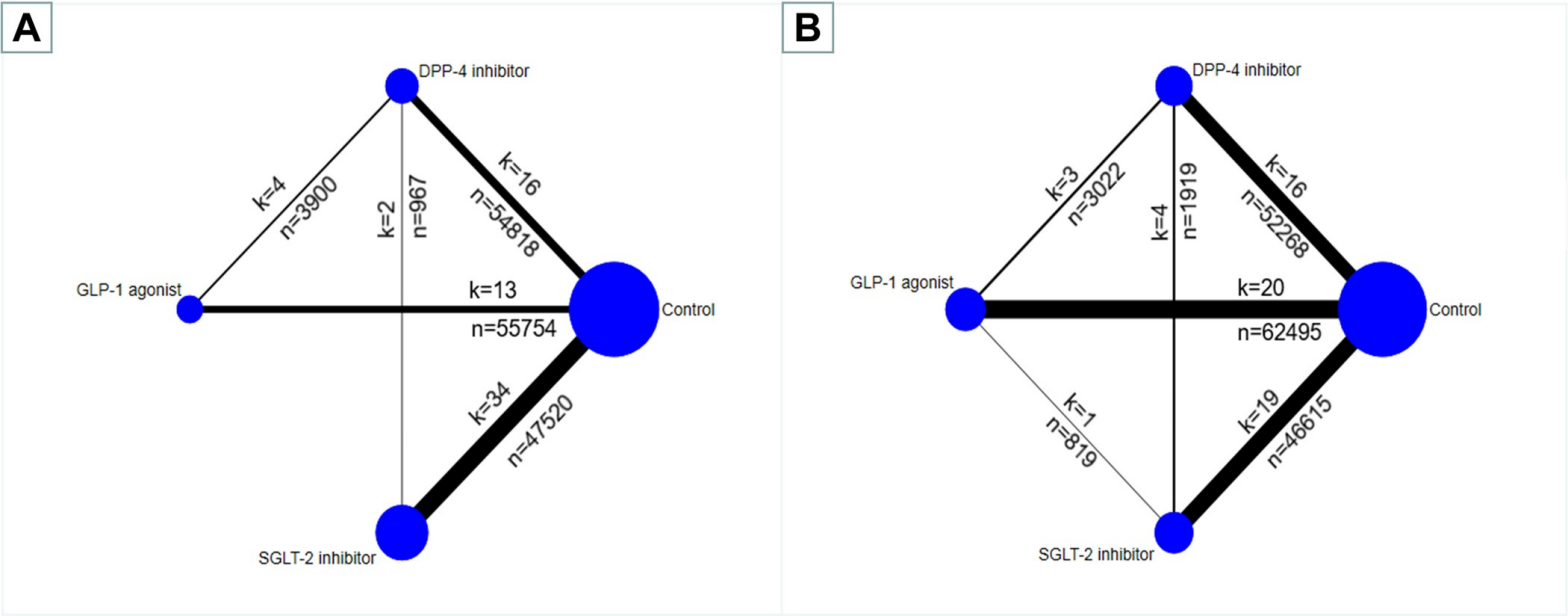
Network meta-analysis of eligible comparisons for composite renal events (A) and AKI events (B).

**Fig 1** shows the study selection process. We retrieved 17 445 studies, of which 98 articles enrolling 186 335 participants were included for the network meta-analysis. For direct comparisons, 39 trials compared an SGLT-2 inhibitor with control, 25 trials compared a GLP-1 agonist with control, 22 trials compared a DPP-4 inhibitor with control, and 12 trials compared between drug classes. Of the 98 included studies, 69 were designed as composite renal outcome trials, and 63 were designed as acute kidney injury outcome trials (**Appendix 4 in S1 Text**). **Fig 2** shows the network of eligible comparisons for composite renal events and acute kidney injury events.

**Appendix 2 in S1 Text** shows the characteristics of the studies. For composite renal outcome trials, the mean sample size was 2467 (range, 47–17, 160), and the mean duration of follow-up ranged between 12 and 302.4 weeks (median, 52; interquartile range, 24–104). The participants’ mean age was 60.4 years, with a median duration of diabetes of 10.97 years.The mean baseline glycated hemoglobin level was 8.13%, and the mean body mass index was 31.19 kg/m^2^. The baseline characteristics of studies were deemed sufficiently similar based on sex, age, hemoglobin A1c (HbA1c) levels, and body mass index (BMI) to permit network comparison.

### Risk of bias of included trials

Of the 98 included studies, 45 (45.9%) were at low risk of bias across all domains. 17 (17.3%) reported adequate random sequence generation; 37 (37.8%) reported adequate allocation concealment. 6 (6.1%) were at high risk of bias for blinding and 17 (17.3%) for attrition bias (**Appendix 3 in S1 Text**). All six high-risk blind bias trials are open-label, and there are deviations from intended interventions in all of them. All of the trials were funded by industry.

### Results of direct comparison by meta-analysis

The analyses of composite renal events included 69 studies that had enrolled 162,959 participants who reported at least one event in any group. In all, there were 9163 events: 1342 (4.9%) of 27 208 participants treated with SGLT-2 inhibitors, 1947 (6.3%) of 30 803 treated with GLP-1 agonists, 1079 (3.7%) of 29 557 treated with DPP-4 inhibitors, and 4795 (6.4%) of 75 391 in the control groups. The results of the pairwise meta-analysis are presented in **Appendix 4 in S1 Text**. GLP-1 agonists were significantly associated with a lower risk of composite renal events than the control groups (OR 0.84, 95% CI 0.78–0.89, *I*^2^ = 0.0%). However, there were no significant differences found between DPP-4 inhibitors and SGLT2 inhibitors and the control groups (OR 1.05, 95% CI 0.96–1.15, *I*^2^ = 0.0%) and (OR 0.88, 95% CI 0.68–1.14, *I*^2^ = 70.1%), respectively. GLP-1 agonists (OR 0.87, 95% CI 0.21–3.65, *I*^2^ = 0.0%) and SGLT-2 inhibitors (OR 1.23, 95% CI 0.61–2.51, *I*^2^ = 0.0%) had no significant difference compared with the DPP-4 inhibitors. Overall, there was no evidence of significant heterogeneity observed, with one exception found between SGLT2 inhibitors and the control groups (*I*^2^ = 70.1%). The heterogeneity was reduced when we excluded one large study [18] and an outlying study [19] (*I*^2^ = 39.3%), but the association was not substantially altered (OR 0.85, 95% CI 0.68 to 1.06).

The analyses of AKI events included data from 63 trials reporting 2002 events among 167,584 patients. The results of the pairwise meta-analysis are presented in **Appendix 4 in S1 Text**. SGLT2 inhibitors were significantly associated with a lower risk of acute kidney injury events than the control groups (OR 0.75, 95% CI 0.65–0.86, *I*^2^ = 0.0%). No statistically significant difference was observed in other head-to-head comparisons. Overall, there was no evidence of significant heterogeneity observed (*I*^2^ = 0.0%).

### Network meta-analysis and drug class rankings

**Fig 3:**
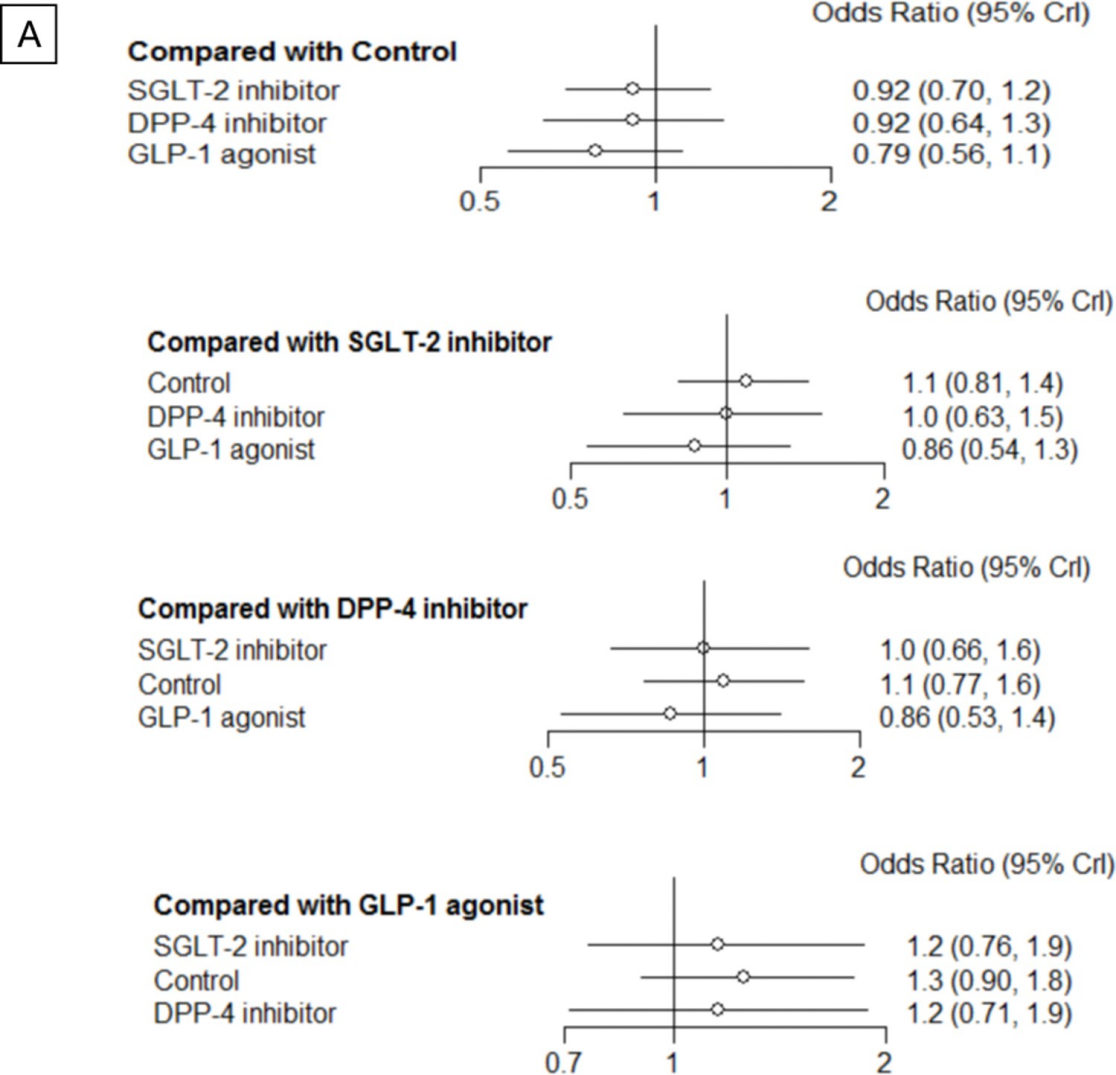
Forest plot for composite renal events (69 trials, 9163 events, 162,959 patients, *I*^2^ = 10%).

For our primary outcome, the network meta-analysis revealed no significant difference between drug classes **(Fig 3).** According to the network contribution plots (**Appendix 3 in S1 Text**), the comparison of placebo versus SGLT-2 inhibitors and GLP-1 agonists had the major impact in entire networks, with 33.7% and 32.8% for composite renal events, respectively.

**Fig 4:**
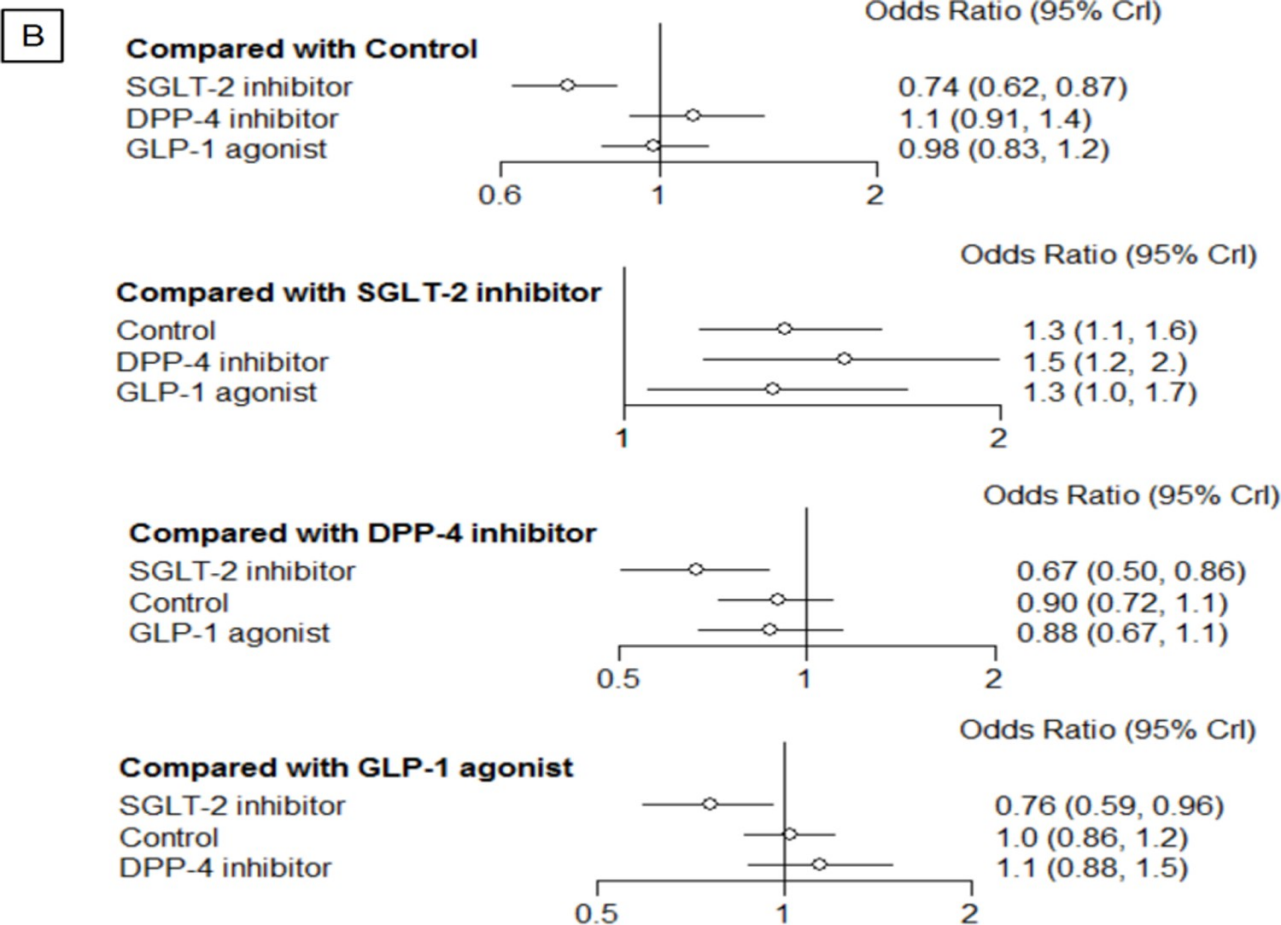
Forest plot for acute kidney injury events (61 trials, 2002 events, 167,584 patients, *I* ^2^ = 15%).

Network meta-analysis suggested that, when compared with the control groups (OR 0.74, 95% CI 0.62–0.87), GLP-1 agonists (OR 0.76, 95% CI 0.59–0.96) and with DPP-4 inhibitors (OR 0.67, 95% CI 0.50–0.86), SGLT-2 inhibitors were associated with a lower risk of acute kidney injury events **(Fig 4)** The comparison of control groups versus SGLT-2 inhibitors, DPP-4 inhibitors and GLP-1 agonists had the major contribution in entire networks, with 32.9%, 32.1% and 32.8% for AKI events, respectively (**Appendix 3 in S1 Text).**

**Fig 5:**
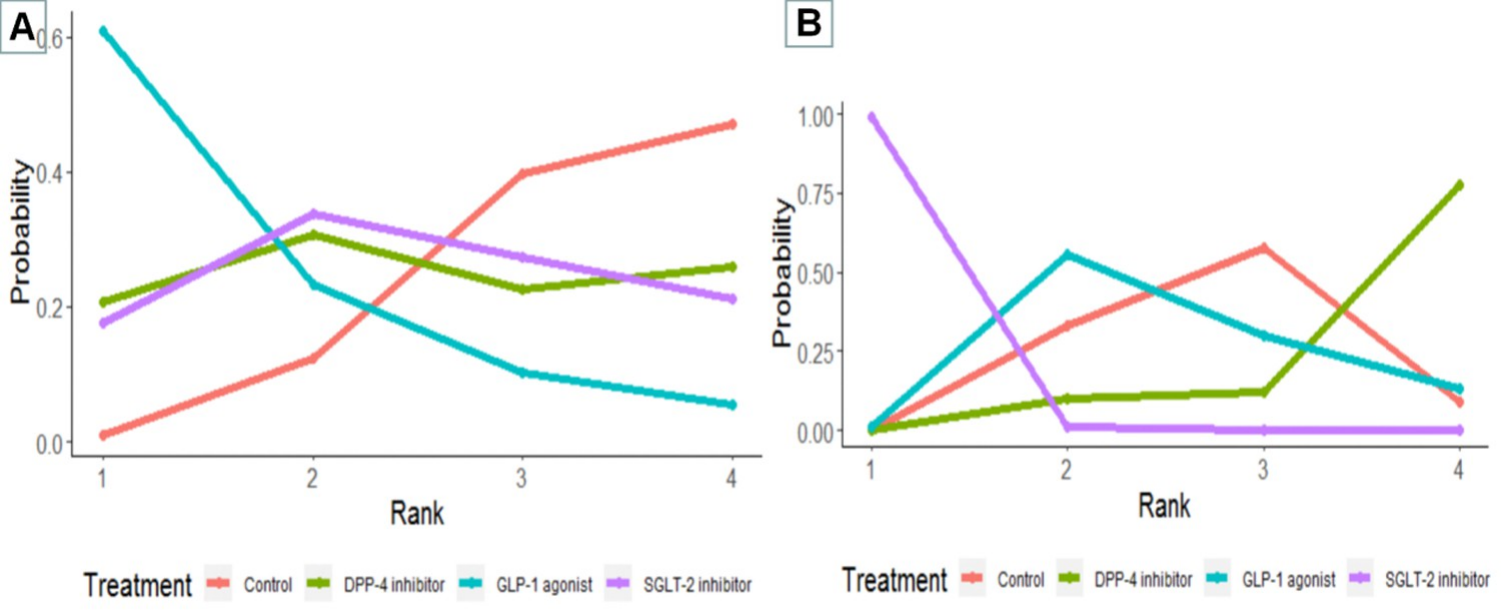
Ranking plots. The higher ranking probabilities indicate a lower risk of composite renal events (A) or acute kidney injury events risk (B). Abbreviation: Control represents either placebo or no treatment; DPP-4, dipeptidyl peptidase 4; GLP-1, glucagon-like peptide 1; and SGLT-2, sodium-glucose cotransporter 2.

We estimated the ranking probabilities of each treatment according to SUCRA, GLP-1 agonists were most likely to rank best for reducing composite renal events, with a probability of 80%, followed by SGLT-2 inhibitors and DPP-4 inhibitors, both with a probability of 49%. For AKI events, SGLT-2 inhibitors were most likely to rank best **(Table 1 and Fig 5**).

**Table 1:** Ranking based on simulations for outcomes.

Outcome	Statistics	SGLT-2 inhibitor	Control	DPP-4 inhibitor	GLP-1 agonist
Composite renal events	SUCRA	0.49	0.22	0.49	0.80
	Rank	2	4	3	1
Acute kidney injury events	SUCRA	1.00	0.41	0.11	0.48
	Rank	1	3	4	2

The higher ranking probabilities indicate a lower risk of composite renal events or acute kidney injury events risk.

Abbreviation: SUCRA: surface under the cumulative ranking curve; Control represents either placebo or no treatment; DPP-4, dipeptidyl peptidase 4; GLP-1, glucagon-like peptide 1; and SGLT-2, sodium-glucose cotransporter 2.

### Transitivity, inconsistency and heterogeneity

We found that the mean BMI, mean age, mean baseline HbA1c, and mean duration of diabetes were all reasonably similar across treatment comparisons when we used box plots to measure transitivity **(Appendix 6 in S1 Text)**. The global inconsistency test found no significant difference between the consistency and inconsistency models for two outcomes (p = 0.902 for composite renal events and p = 0.626 for acute kidney injury events in **Appendix 7 in S1 Text**). After constructing the node-splitting models, we found that no significant inconsistency existed in all networks except for acute kidney injury events (Control vs SGLT-2i and DPP-4i vs SGLT-2i; **Appendix 7 in S1 Text**). Heterogeneity (global *I*^2^)was low for both our primary and secondary outcomes (10% and 15%, respectively). At visual inspection, comparison-adjusted funnel plots for composite renal outcomes appeared to be symmetric, indicating that there was no significant risk of publication bias in our included studies. However, the funnel plots suggest that there might be small-study effects for AKI events (**S3 Fig and Appendix 3 in S1 Text**).

### Sensitivity analysis, subgroup analysis and meta-regression analysis

We did a series of subgroup analyses for our outcomes (**Appendix 8 in S1 Text**). The effect estimate for composite renal outcome events did not change between the CVD and non-CVD groups, nor did the ACEi/ARB treatment and non-ACEi/ARB treatment groups. However, in our cardiovascular outcome group and CKD group, SGLT-2 inhibitors and GLP-1 agonists both significantly lowered the renal composite outcome risk compared to the control groups. Subgroup analyses found that SGLT-2 inhibitors were more effective than the control groups and DPP-4 inhibitors in lowering AKI events in patients with CVD, CKD, and ACEi/ARB treatment. Sensitivity analyses did not affect the associations of SGLT-2 inhibitors with reduced AKI events compared with the control groups, DPP-4inhibitors, and GLP-1 agonists (**Appendix 9 in S1 Text**). Network meta-regression indicated that covariates had no effect on outcome except for DPP-4 inhibitor versus control groups, and that composite renal outcome events would be influenced by eGFR at baseline (**Appendix 10 in S1 Text**).

### GRADE assessment on quality of evidence

According to GRADE, the quality of evidence ranged from very low to moderate, but was classified as low or moderate in the majority of comparisons. The quality was low for GLP-1 agonists versus control groups, moderate for DPP-4 inhibitors versus control groups, and very low for SGLT-2 inhibitors versus control groups in composite renal events. For AKI events, the quality of SGLT-2 inhibitors against control groups, DPP-4 inhibitors, and GLP-1 agonists was low, low, and moderate, respectively. The overall ranking of treatments for our primary and secondary outcomes had a moderate quality of evidence (**Appendix 11 in S1 Text**).

## Discussion

Our study is the first network meta-analysis to address the effects of SGLT-2 inhibitors, GLP-1 agonists, and DPP-4 inhibitors on kidney outcomes in participants with type 2 diabetes. Our findings can be summarized as follows: First, GLP-1 receptor agonists were most likely ranked best among the three drugs in reducing composite renal events. However, we did not find significant differences between drug classes regardless of baseline factors. Second, the SGLT-2 inhibitors were associated with a lower risk of AKI events than DPP-4 inhibitors, GLP-1 agonists, and control groups.

To date, no randomized clinical trials with kidney outcomes have directly compared the efficacy of these three new antidiabetic drug classes. Although both SGLT-2 inhibitors and GLP-1 receptor agonists have been reported to reduce kidney events in several trials, which one of them yields more renal benefits has not been clear. Moreover, these renal outcome studies were based on large cardiovascular outcome trials (CVOTs). Most participants were people with established cardiovascular diseases or cardiovascular risk factors, without considering baseline renal function. Therefore, the applicability of this conclusion is limited. Moreover, if the population without these risks, the effect of these three classes of glucose-lowering drugs on the renal outcome is difficult to determine.

Our meta-analysis found that SGLT-2 inhibitors were similar to placebo or no treatment concerning kidney events regardless of baseline factors. However, in our cardiovascular outcome subgroup, SGLT-2 inhibitors are superior to the other two classes in terms of renal endpoints. These findings are consistent with recent meta-analysis of CVOTs on this drug class [20, 21]. For particular outcomes, the clinical effects of SGLT-2 inhibitors depend on the patient population in which they are used. The current meta-analysis [22] showed that SGLT2 inhibitors reduced the risk of renal disease progression (including worsening eGFR, end-stage renal disease, or renal death) by 45%, with a similar benefit in those with and without atherosclerotic cardiovascular disease. However, with more severe kidney disease at baseline, fewer renal disease progression reductions were found.

In our pairwise meta-analysis, GLP-1 agonists were associated with reductions in composite renal events compared with the control groups. In our network meta-analysis, the advantage of GLP-1 agonists was not significant. This conclusion is consistent with some current research. A recent meta-analysis [14] of 7 trials with a combined total of 56 004 participants demonstrated that GLP-1 receptor agonist treatment reduced a composite renal outcome, including development of new-onset macroalbuminuria, a decline in estimated glomerular filtration rate (or increase in creatinine), progression to end-stage kidney disease, or death attributable to kidney causes, compared with placebo (HR, 0.83; 95% CI, 0.78–0.89). In another meta-analysis [6] of clinical trials with renal outcomes, incorporating data from 42,920 patients, showed that GLP-1 receptor agonists reduced the risk of kidney disease progression, including macroalbuminuria, compared with placebo (HR, 0.82; 95% CI, 0.75–0.89). But these effects were driven by a reduction in urinary albumin excretion, and the benefit based on a decline in eGFR (or increase in creatinine) was not significant. Although albuminuria is a recognized biomarker reflecting diabetic nephropathy risk, it represents a surrogate marker that may even be absent in patients with reduced eGFR [23]. Thus, eGFR reduction has become a more meaningful endpoint and is being used for kidney outcomes in ongoing diabetes trials [24].

Despite extensive exploratory analyses, the exact pathophysiological explanations of how SGLT-2 inhibitors and GLP-1 agonists exert their favorable effects are still unclear [25]. The natriuresis and inhibition of tubuloglomerular feedback by SGLT-2 inhibitors may play a central role and explain the observed reduction in delayed diabetic kidney disease progression [26].

In our meta-analysis, we did not find a beneficial effect of DPP-4 inhibitors on renal outcomes. This conclusion is similar to the current study. In the latest CARMELINA [27] trial, 74% of patients had prevalent chronic kidney disease (defined as eGFR <60 mL/min/1.73 m^2^ and/or UACR >300 mg/g creatinine), 43% had an eGFR below 45 mL/min/1.73 m^2^, and 15.2% had an eGFR below 30 mL/min/1.73 m^2^. The study population was at high renal risk. In this trial, there was no significant benefit of linagliptin compared with placebo for the kidney composite outcome, but there was a significant reduction in microvascular events driven primarily by the reduction in albuminuria progression.

There is a lack of research on the renal outcomes of these new antidiabetic drug classes in type 2 diabetic patients with CKD. Our study attempted a subgroup analysis based on the mean eGFR and the proportion of chronic kidney disease. In our pre-existing CKD subgroup analysis, the use of SGLT-2 inhibitors or GLP-1 agonists was associated with reductions in renal events compared with placebo or no treatment. No matter in the overall analysis, Non-cardiovascular outcome subgroup, and non-CKD subgroup analysis, no drug class was associated with a reduction in composite renal events compared with the control groups. However, GLP-1 agonists were most likely ranked best among them. The relative benefit of these three agents on renal outcomes in different patient populations remains undefined. The effect on type 2 diabetes patients with or without CKD needs to be further investigated.

Compared with control groups, SGLT-2 inhibitors were associated with a lower risk of AKI events compared with other groups. These findings are consistent with a recent meta-analysis [28, 29]. In our meta-analysis, we found that GLP-1 receptor agonists and DPP-4 inhibitors were not associated with an increased risk of AKI events. This conclusion is similar to recent meta-analyses of patients who have established cardiovascular diseases or cardiovascular risk factors [30].

### Limitations

Our study has some limitations, which are worth further discussion. First, there were no head-to-head kidney outcomes trials that directly compared the three new classes of antidiabetic drugs. The comparative effects were generated with indirect evidence. The clinical effect of the drug was evaluated by drug class rather than by individual drug type. Although this increases the power to detect treatment effects, and there could be different within-class treatments. Second, Some of the data for composite renal events come from adverse event reporting rather than trial data, of the 69 studies with composite renal outcome events, 11 (15.9%) were directly measured and 58 (84.1%) were evaluated based on adverse reports. In comparison to 8.04 percent in direct measurement trials, composite renal events accounted for 1.87 percent of adverse reports. None of the trials included were systematically designed to investigate AKI events. The majority of AKI event data comes from adverse event reporting rather than trial data. Such constraints reduce the validity of our meta-analysis. Third, there were differences in background treatments, patient characteristics, and outcome evaluation among studies that might contribute to heterogeneity. Finally, the limitations of our meta-analysis include the absence of patient-level data, restriction of subgroup analyses to the primary outcome, and our ability to examine only the secondary endpoints of particular interest reported by the investigators of the included trials.

## Conclusions

In this network meta-analysis, although none of the three new antidiabetic drug classes reduced the composite renal events in participants with type 2 diabetes, GLP-1 receptor agonists may be more effective. The use of SGLT-2 inhibitors was associated with a lower AKI event risk than DPP-4 inhibitors, GLP-1 agonists, placebo, or no treatment.

## Supporting information

S1 Checklist (DOCX)

S1 Text (DOC)
