# Supplementary material for: Association between use of sodium-glucose cotransporter 2 inhibitors, glucagon-like peptide 1 agonists, and dipeptidyl peptidase 4 inhibitors with kidney outcomes in patients with type 2 diabetes: A systematic review and network meta-analysis
Source: PLoS One. 2022 Apr 14;17(4):e0267025. doi: 10.1371/journal.pone.0267025 (PMC9009659; doi:10.1371/journal.pone.0267025)
Supplement: S1 Text — (DOC) [file pone.0267025.s002.doc]

**Contents of supplementary appendix**

Appendix 1 2

Search strategy

Appendix 2 7

Characteristics of included studies

Appendix 3 32

Risk of bias assessment

Appendix 4 40

Pairwise meta-analysis of the effects of glucose-lowering drugs on the risk of composite renal events and acute kidney injury events

Appendix 5 42

Deviance information criterion for model selection

Appendix 6 42

Assessment of transitivity

Appendix 7 43

Evaluation of inconsistency

Appendix 8 44

Subgroup network meta-analyses

Appendix 9 47

Sensitivity analyses

Appendix 10 48

Meta-regression for network meta-analyses

Appendix 11 50

GRADE for outcomes

Appendix 12 56

Drug doses and the code of network meta-analysis for main results in R software

Appendix 13 59

Extract form

Appendix 14 63

References for included trials

**Appendix 1. Search strategy**

**Supplemental Table 1**. Search Strategy

| **Data source** | **Search terms** |
| --- | --- |
| **PubMed (SGLT2 inhibitor)** | #1 Diabetes Mellitus, Type 2 OR Type 2 Diabetes Mellitus OR Type 2 Diabetes OR type ii diabetes  #2 Sodium glucose co-transporter OR Sodium glucose cotransporter 2 inhibitor OR sodium glucose transporter 2 inhibitor OR sodium glucose transporter ii inhibitor OR SGLT2 inhibitor OR SGLT2 OR SGLT-2 OR SGLT 2  #3 Tofogliflozin OR Apleway OR Deberza OR CSG452 OR tofogliflozin hydrate OR CSG452 OR tofogliflozin anhydrous  #4 Empagliflozin OR Jardiance OR BI 10773 OR BI10773 OR BI-10773  #5 Dapagliflozin OR Farxiga OR Forxiga OR BMS 512148 OR BMS512148 OR BMS-512148  #6 Canagliflozin OR Invokana OR Canagliflozin Hemihydrate OR Canagliflozin, Anhydrous  #7 Sotagliflozin OR LX4211 OR LX-4211  #8 luseogliflozin OR Lusefi OR TS 071 OR TS071 cpd OR TS-071  #9 Ipragliflozin OR Suglat OR ASP1941 OR ASP-1941  #10 remogliflozin  #11 sergliflozin  #12 ertugliflozin OR PF 04971729 OR PF-04971729  #13 Randomized Controlled Trial[Publication Type] OR randomized[Title/Abstract] OR placebo[Title/Abstract]  #14 OR#2- #12  #15 #1 AND #13 AND #14 |
| **CENTRAL (SGLT2 inhibitor)** | ○ Diabetes Mellitus, Type 2 or Type 2 Diabetes Mellitus or Type 2 Diabetes or type ii diabetes  ○ (Sodium glucose co-transporter* or Sodium glucose cotransporter 2 inhibitor* or sodium glucose transporter 2 inhibitor* or sodium glucose transporter ii inhibitor* or SGLT2 inhibitor* or SGLT2 or SGLT-2 or SGLT 2)  OR  ○ Tofogliflozin or Empagliflozin or Dapagliflozin or Canagliflozin or Sotagliflozin or Luseogliflozin or Lpragliflozin or Remogliflozin or Ertugliflozin |
| **Embase (SGLT2 inhibitor)** | #1 (Diabetes Mellitus, Type 2 or Type 2 Diabetes Mellitus or Type 2 Diabetes or type ii diabetes).af  #2 (Sodium glucose co-transporter).af or (Sodium glucose cotransporter 2 inhibitor* or sodium glucose transporter 2 inhibitor* or sodium glucose transporter ii inhibitor* or SGLT2 inhibitor* or SGLT2 or SGLT-2 or SGLT 2).af  #3 (Tofogliflozin or Empagliflozin or Dapagliflozin or Canagliflozin or Sotagliflozin or Luseogliflozin or Lpragliflozin or Remogliflozin or Ertugliflozin ).af  #4 (randomi*ed controlled trial or randomi*ed trial).af  #5 #2 or #3  #6 #1 and #4 and #5 |
| **PubMed (GLP-1 analogue)** | #1 Diabetes Mellitus, Type 2 OR Type 2 Diabetes Mellitus OR Type 2 Diabetes OR type ii diabetes  #2 glucagon-like peptide-1 receptor agonists OR glucagon-like peptide 1  receptor inhibitor OR glucagon-like peptide 1 receptor agonist OR glucagon like peptide 1 inhibitor OR glucagon-like peptide 1 agonist OR GLP-1 receptor inhibitor OR GLP-1 receptor agonist OR GLP-1 inhibitor OR GLP-1 agonist  #3 Albiglutide OR Eperzan OR Tanzeum  #4 Dulaglutide OR LY 2189265 OR LY-2189265 OR LY2189265 OR Trulicity  #5 Semaglutide  #6 Liraglutide OR Victoza OR Saxenda OR NN 2211 OR 2211, NN OR NN2211 OR NN-2211  #7 Lixisenatide OR Adlyxin OR AQVE-10010 OR ZP10A peptide OR ZP 10 OR ZP-10 OR Lyxumia OR AVE 010 OR AVE 0010 OR AVE0010 OR AVE-0010  #8 Taspoglutide  #9 Exenatide OR Bydureon OR ITCA 650 OR AC 2993 LAR OR Exendin-4 OR Ex4 Peptide OR Peptide, Ex4 OR Exendin 4 OR Byetta OR AC 2993  #10 Randomized Controlled Trial[Publication Type] OR randomized[Title/Abstract] OR placebo[Title/Abstract]  #11 OR#2- #9  #12 #1 AND #10 AND #11 |
| **CENTRAL (GLP-1 analogue)** | ○ Diabetes Mellitus, Type 2 or Type 2 Diabetes Mellitus or Type 2 Diabetes or type ii diabetes  ○ (glucagon-like peptide-1 receptor agonist* or glucagon-like peptide 1  receptor inhibitor* or glucagon-like peptide 1 receptor agonist* or glucagon like peptide 1 inhibitor* or glucagon-like peptide 1 agonist* or GLP-1 receptor inhibitor* or GLP-1 receptor agonist* or GLP-1 inhibitor* or GLP-1 agonist*)  OR  ○ Albiglutide or Dulaglutide or Semaglutide or Liraglutide or Lixisenatide or Taspoglutide or Exenatide |
| **Embase (GLP-1 analogue)** | #1 (Diabetes Mellitus, Type 2 or Type 2 Diabetes Mellitus or Type 2 Diabetes or type ii diabetes).af  #2 (glucagon-like peptide-1 receptor agonist).af or (glucagon-like peptide 1 receptor inhibitor* or glucagon-like peptide 1 receptor agonist* or glucagon like peptide 1 inhibitor* or glucagon-like peptide 1 agonist* or GLP-1 receptor inhibitor* or GLP-1 receptor agonist* or GLP-1 inhibitor* or GLP-1 agonist*).af  #3 (Albiglutide or Dulaglutide or Semaglutide or Liraglutide or Lixisenatide or Taspoglutide or Exenatide).af  #4 (randomi*ed controlled trial or randomi*ed trial).af  #5 #2 or #3  #6 #1 and #4 and #5 |
| **PubMed (DPP-4 inhibitor)** | #1 Diabetes Mellitus, Type 2 OR Type 2 Diabetes Mellitus OR Type 2 Diabetes OR type ii diabetes  #2 Dipeptidyl peptidase IV inhibitor OR Dipeptidyl Peptidase IV Inhibitors OR Inhibitors, Dipeptidyl-Peptidase IV OR Gliptins OR Dipeptidyl-Peptidase 4 Inhibitors OR Dipeptidyl Peptidase 4 Inhibitors OR DPP4 inhibitor OR DPP 4 inhibitor OR DPP IV inhibitor  #3 Alogliptin OR nesina OR SYR 322 OR SYR322 OR SYR-322  #4 Anagliptin OR anagliptin hydrochloride  #5 Gemigliptin  #6 Linagliptin OR BI 1356 OR 1356, BI OR BI1356 OR BI-1356 OR Tradjenta OR Trajenta  #7 Omarigliptin OR Omarigliptin OR MK-3102  #8 Saxagliptin OR Onglyza OR BMS 477118 OR BMS477118 OR BMS-477118  #9 Sitagliptin OR Phosphate, Sitagliptin OR Sitagliptin Phosphate Monohydrate OR Monohydrate, Sitagliptin Phosphate OR Phosphate Monohydrate, Sitagliptin OR Sitagliptin Monophosphate Monohydrate OR Monohydrate, Sitagliptin Monophosphate OR Monophosphate Monohydrate, Sitagliptin OR MK 0431 OR 0431, MK OR MK0431 OR MK-0431 OR Sitagliptin Phosphate Anhydrous OR Anhydrous, Sitagliptin Phosphate OR Phosphate Anhydrous, Sitagliptin OR Sitagliptin OR Januvia  #10 Teneligliptin  #11 Vildagliptin OR NVP-LAF237 OR NVP LAF237 OR Galvus  #12 Randomized Controlled Trial[Publication Type] OR randomized[Title/Abstract] OR placebo[Title/Abstract]  #13 OR#2- #11  #14 #1 AND #12 AND #13 |
| **CENTRA (DPP-4 inhibitor)** | ○ Diabetes Mellitus, Type 2 or Type 2 Diabetes Mellitus or Type 2 Diabetes or type ii diabetes  ○ (Dipeptidyl peptidase IV inhibitor* or Inhibitors, Dipeptidyl-Peptidase IV or Gliptins or Dipeptidyl-Peptidase 4 Inhibitor* or Dipeptidyl Peptidase 4 Inhibitor* or DPP4 inhibitor or DPP 4 inhibitor or DPP IV inhibitor)  OR  ○ Alogliptin or Anagliptin or Gemigliptin or Linagliptin or Omarigliptin or Saxagliptin or Sitagliptin or Teneligliptin or Vildagliptin |
| **Embase (DPP-4 inhibitor)** | #1 (Diabetes Mellitus, Type 2 or Type 2 Diabetes Mellitus or Type 2 Diabetes or type ii diabetes).af  #2 (Dipeptidyl peptidase IV inhibitor).af or (Inhibitors, Dipeptidyl-Peptidase IV or Gliptins or Dipeptidyl-Peptidase 4 Inhibitor* or Dipeptidyl Peptidase 4 Inhibitor* or DPP4 inhibitor or DPP 4 inhibitor or DPP IV inhibitor).af  #3 (Alogliptin or Anagliptin or Gemigliptin or Linagliptin or Omarigliptin or Saxagliptin or Sitagliptin or Teneligliptin or Vildagliptin).af  #4 (randomi*ed controlled trial or randomi*ed trial).af  #5 #2 or #3  #6 #1 and #4 and #5 |

**Appendix 2. Characteristics of included studies**

**Supplemental Table 2**. Baseline Characteristics

| **Trial** | **Total randomised** | **Drug** | **Comparator** | **Background treatments** | **Mean Age (years)** | **Male (%)** | **Mean eGFR: (ml/min/1.73 m2 )** | **Pre-existing CKD (%)** | **Pre-existing CVD (%)** | **Follow up (week)** | **Mean HbA1c (%)** | **Mean Duration of diabetes (years)** | **Background**  **ACEi/ARB (%)** | **Mean BMI (kg/m2)** | **Outcomes reported** | **Data source of outcome** |
| --- | --- | --- | --- | --- | --- | --- | --- | --- | --- | --- | --- | --- | --- | --- | --- | --- |
| **SGLT-2 Inhibitor vs. Control (39studies)** | | | | | | | | | | | | | | | |  |
| Stenl ¨of 2013^1^ | 584 | Canagliflozin | Placebo | drug-na¨ıve | 55.4±10.6 | 44.20 | NR | NR | NR | 26 | 8.0±1.0 | 4.3±4.4 | NR | 31.6± 6.2 | Renal failure acute | Based on prespecified SAE item from MedDRA 14.0 (Clinitrial trial registration) |
| Yale 2014^2^ | 269 | Canagliflozin | Placebo | SU/INS± OAD | 68.5±8.28 | 60.6 | 39.4±6.9^a^ | 100^a^ | 54.6 | 52 | 8.0±0.9 | 16.3±8.5 | 87.4 | 33.0±6.2 | Decreased renal function  (e.g. specific terms of renal impairment and blood creatinine increased); Proportion of patients with progression in albuminuria | Publications (AEs of special interest Renal events) |
|  |  |  |  |  |  |  |  |  |  |  |  |  |  |  | Renal failure acute | Based on prespecified SAE item from MedDRA 15.0 (Clinitrial trial registration) |
| Bode 2015^3^ | 714 | Canagliflozin | Placebo | INS ± OAD | 63.6±6.24 | 55.50 | 77.5±16.6 | NR | NR | 104 | 7.7±0.8 | 11.7±7.5 | NR | 31.6±4.6 | Renal impairment | Based on prespecified SAE item from MedDRA 16.0 (Clinitrial trial registration) |
| CREDENCE (Mahaffey) 2019^4^ | 4401 | Canagliflozin | Placebo | INS ± OAD | 63±9.2 | 66.10 | 56.14±18.21^b^ | 95.2^b^ | NR | 136.6 | 8.3±1.3 | 15.8±8.7 | 100 | 31.35±6.15 | End-stage kidney disease, doubling of serum creatinine  level, or renal death | Publications |
|  |  |  |  |  |  |  |  |  |  |  |  |  |  |  | Acute Kidney Injury | Based on prespecified SAE item from MedDRA 21.0 (Clinitrial trial registration) |
| CANVAS (Perkovic) 2018^5^ | 10142 | Canagliflozin | Placebo | INS ± OAD | 63.3±8.3 | 64.18 | 76.46±20.50^b^ | 75.58^b^ | 65.6 | 188.2 | 8.23±0.9 | 13.58±7.45 | 80.02 | 31.97±5.94 | Composite outcome (40% reduction in eGFR, ESKD, death from renal causes, or new-onset macroalbuminuria) | Publications |
|  |  |  |  |  |  |  |  |  |  |  |  |  |  |  | Acute Kidney Injury | Based on prespecified SAE item from MedDRA 19.1 (Clinitrial trial registration) |
| Rosenstock 2016^6^ | 1186 | Canagliflozin | No treatment | drug-na¨ıve | 55.93±9.74 | 47.68 | 87.67±2.12 | NR | NR | 30 | 8.83±1.17 | 3.17±3.93 | NR | 32.57±5.96 | Renal-related AEs(Includes blood creatinine increased, glomerular filtration rate decreased, and renal impairment) | Publications |
| Qiu 2014^7^ | 279 | Canagliflozin | No treatment | MET | 57.4±9.53 | 46.6 | 85.9±16.6 | NR | NR | 18 | 7.6±0.9 | 7.0±5.8 | NR | 32.5±6.5 | GFR decreased (lead ing to discontinuation ) based on AE reports, and safety laboratory tests | Publications |
| Allegretti 2019^8^ | 312 | Bexagliflozin | Placebo | INS ± OAD | 69.6±8.32 | 62.82 | 45.11±8.32^a^ | 100^a^ | NR | 24 | 7.98±0.798 | 15.91±9.082 | 73.7 | 30.20±5.87 | Acute kidney injury (All AKI events were stage 1 AKI) | Publications |
| VERTIS RENAL (Grunberger) 2018^9^ | 468 | Ertugliflozin | Placebo | INS ± OAD | 67.3±8.6 | 49.50 | 46.6±8.8^a^ | 100^a^ | 49.7^d^ | 52 | 8.15±0.93) | 14.2±8.5 | NR | 32.5±6.1 | Renal-related AEs (defined according to a standard MedDRA composite query of acute kidney injury, acute prerenal failure, or renal impairment) | Publications |
|  |  |  |  |  |  |  |  |  |  |  |  |  |  |  | Acute kidney injury | Based on prespecified SAE item from MedDRA 19.0 (Clinitrial trial registration) |
| Haneda 2016^10^ | 145 | Luseogliflozin | Placebo | OAD | 68±8.9 | 76.60 | 52.1±9.0 | 54.2 | NR | 24 | 7.71±0.67 | 11.1±8.3 | NR | 25.58±4.09 | AE related to renal function (e.g. blood Cr increased and GFR decreased) based on prespecified AE item from MedDRA | Publications |
| EMPA-REG OUTCOME  (Monteiro) 2019^11^ | 7020 | Empagliflozin | Placebo | INS ± OAD | 63.1±8.6 | 71.50 | 74.05±21.41^a^ | 77.72^a^ | Based on prespecified SAE item from MedDRA 15.0 (Clinitrial trial registration) | 192 | 8.1±0.85 | NR | 80.71 | 30.6 ±5.3 | Incident or worsening nephropathy; Progression to macroalbuminuria (UACR >300 mg/g) | Publications |
|  |  |  |  |  |  |  |  |  |  |  |  |  |  |  | Acute renal failure (Based on prespecified AE items from MedDRA18.0) | Publications |
| Barnett 2014^12^ | 741 | Empagliflozin | Placebo | INS ± OAD | 63.9±8.8 | 58.30 | 71.6±10.6^a^ | 100^a^ | NR | 65.43 | 8.0±0.79 | NR | 74 | 30.73±5.48 | Renal failure, renal failure chronic and renal impairment | Based on prespecified SAE item from MedDRA 15.0 (Clinitrial trial registration) |
| Rosenstock 2015^13^ | 494 | Empagliflozin | Placebo | INS ± MET± SU | 58.8±9.9 | 55.9 | NR | NR | NR | 82 | 8.2±0.8 | NR | NR | NR | Renal failure | Based on prespecified SAE item from MedDRA 15.0 (Clinitrial trial registration) |
|  |  |  |  |  |  |  |  |  |  |  |  |  |  |  | Renal failure acute |  |
| Rosenstock 2014^14^ | 563 | Empagliflozin | Placebo | INS ± MET | 56.7±9.5 | 45.50 | 84±16.6^a^ | NR | NR | 52 | 8.34±0.73 | NR | NR | 34.8±4.1 | Renal impairment | Based on prespecified SAE item from MedDRA 16.0 (Clinitrial trial registration) |
|  |  |  |  |  |  |  |  |  |  |  |  |  |  |  | Renal failure acute |  |
| Häring 2014^15^ | 638 | Empagliflozin | Placebo | MET | 55.7±9.9 | 57.00 | 89.0±20.1^a^ | NR | NR | 76 | 7.9±0.85 | NR | NR | 29.2±5.5 | Renal failure | Based on prespecified SAE item from MedDRA 16.0 (Clinitrial trial registration) |
|  |  |  |  |  |  |  |  |  |  |  |  |  |  |  | Renal failure acute |  |
| Haering 2015^16^ | 666 | Empagliflozin | Placebo | MET±SU | 57.1±9.2 | 51.00 | 87.2±21.5^a^ | NR | NR | 76 | 8.10±0.83 | NR | NR | 28.2±5.3 | Renal failure | Based on prespecified SAE item from MedDRA 16.0 (Clinitrial trial registration) |
|  |  |  |  |  |  |  |  |  |  |  |  |  |  |  | Renal failure acute |  |
| Roden 2015^17^ | 899 | Empagliflozin | Sitagliptin/Placebo | drug-na¨ıve | 55±11.0 | 61.30 | 87.4±18.2^a^ | NR | NR | 76 | 7.88±0.82 | NR | NR | 28.4±5.6 | Renal failure | Based on prespecified SAE item from MedDRA 16.0 (Clinitrial trial registration) |
|  |  |  |  |  |  |  |  |  |  |  |  |  |  |  | Renal failure acute |  |
| Søfteland 2017^18^ | 333 | Empagliflozin | Placebo | MET+LINA | 55.2±9.7 | 59.60 | 92.31±18.03^a^ | 49.24^a^ | NR | 25.14 | 7.97±0.83 | NR | NR | 30.23±5.66 | Renal failure acute | Based on prespecified SAE item from MedDRA 17.1 (Clinitrial trial registration) |
| Kohan 2014^19^ | 252 | Dapagliflozin | Placebo | INS±OAD | 67.0±8.5 | 65.10 | 55.57±9.8 | 100 | NR | 104 | 8.35±1.12 | 16.94±9.57 | NR | NR | Blood creatinine increased, renal impairment and renal failure | Publications (Renal impairment or failure based on adverse event or laboratory abnormalities) |
|  |  |  |  |  |  |  |  |  |  |  |  |  |  |  | Renal failure acute |  |
| Cefalu 2015^20^ | 922 | Dapagliflozin | Placebo | INS±OAD | 62.9±7.32 | 68.30 | NR | 59.5 | 100^e^ | 52 | 8.13±0.832 | 12.45±8.45 | 89.39 | 32.75±6.0 | Events of renal impairment/failure | Publications (Renal impairment/ failure (e.g. decreased renal Cr clearance, renal impairment, acute renal failure, increased blood Cr, decreased GFR) based on prespecified renal impairment/ failure AE items from MedDRA) |
|  |  |  |  |  |  |  |  |  |  |  |  |  |  |  | Acute renal failure |  |
| Leiter 2014^21^ | 964 | Dapagliflozin | Placebo | INS±OAD | 68.3±7.31 | 66.90 | NR | 59.5 | 100 | 52 | 8.06±0.78) | 12.85±8.35) | 83.47 | 32.85±5.50 | Renal impairment or failure | Publications (Renal impairment/failure (e.g. renal impairment, renal failure, and acute renal failure) based on prespecified AE item from MedDRA) |
|  |  |  |  |  |  |  |  |  |  |  |  |  |  |  | Acute renal failure |  |
| Kaku 2014^22^ | 261 | Dapagliflozin | Placebo | drug-na¨ıve | 58.8±9.84 | 59.40 | 67.07±11.58 | 68.97 | 45.59 | 24 | 7.49±0.65 | 4.94±5.87 | NR | 25.39 ±4.296 | Renal impairment (Renal impairment based on laboratory values, calculated Cr clearance, and eGFR) | Publications (AEs of special interest Renal events) |
| DECLARE–TIMI 58 (mosenzon) 2019^23^ | 17160 | Dapagliflozin | Placebo | INS±OAD | 63.9±6.8 | 62.60 | 85.24±15.89^b^ | 52.43^b^ | 40.6 | 219 | 8.3±1.18 | 11.82±7.74 | 81.29 | 32.04±6.05 | Composite renal-specifific outcome (Sustained decrease in eGFR by at least 40% to less than 60 mL/min per 1·73 m², end-stage renal disease, or renal death. eGFR was calculated with the Chronic Kidney Disease Epidemiology Collaboration equation) | Publications |
|  |  |  |  |  |  |  |  |  |  |  |  |  |  |  | Acute kidney injury | Based on prespecified SAE item from MedDRA 21.0 (Clinitrial trial registration) |
| Pollock 2019^24^ | 293 | Dapagliflozin | Placebo | INS±OAD | 64.7±8.5 | 70.65 | 48.94±13.29 | 100 | 48.46% | 28 | 8.51±1.11 | 17.63±8.64 | 98.98 | 30.27±5.44 | AEs of special interest Renal events (Sustained increase (>1·5times increase) in serum creatinine) | Publications |
|  |  |  |  |  |  |  |  |  |  |  |  |  |  |  | Acute kidney injury | Based on prespecified SAE item from MedDRA 21.0 (Clinitrial trial registration) |
| Heerspink 2016^25^ | 356 | Dapagliflozin | Placebo | INS±OAD | 54.96±8.75 | 64.04 | 84.06±20.46^a^ | 100^f^ | NR | 12 | 8.1±0.95 | 8.4±6.18 | 100 | 31.44±5.39 | AEs of special interest Renal events | Publications |
| Bailey 2013^26^ | 409 | Dapagliflozin | Placebo | MET | 53.57±9.87 | 54.28 | NR | NR | NR | 102 | 8.07±0.92 | 6.1±5.43 | NR | 31.47±5.13 | Renal impairment or failure (Renal impairment/failure (e.g. increases in serum Cr >1.5 timesthe baseline value or attaining an absolute value of 221 umol/l) (defined by a prespecified list)) | Publications |
|  |  |  |  |  |  |  |  |  |  |  |  |  |  |  | Acute renal failure | Based on prespecified SAE item from MedDRA 13.0 (Clinitrial trial registration) |
| Bailey 2012^27^ | 136 | Dapagliflozin | Placebo | drug-na¨ıve | 52.4±11.31 | 50.74 | NR | NR | NR | 24 | 7.85±1.07 | 1.25±2.67 | NR | 31.72±5.34 | Renal impairment (Renal impairment based on laboratory values, calculated Cr clearance, and eGFR) | Publications |
| Bailey 2015^28^ | 209 | Dapagliflozin | Placebo | drug-na¨ıve | 51.96±10.38 | 45.93 | NR | NR | NR | 102 | 7.90±0.92 | 1.1±2.17 | NR | 32.61±5.28 | Renal impairment/failure | Publications (AEs of special interest Renal events) |
| Araki 2016^29^ | 182 | Dapagliflozin | Placebo | INS±DPPIV | 58.0±9.8) | 70.90 | NR | 71.98 | NR | 16 | 8.34± 0.849 | 14.97±8.952 | 41 | 26.64±4.510 | Renal impairment/failure | Publications (AEs of special interest Renal events) |
| Wilding 2009^30^ | 47 | Dapagliflozin | Placebo | INS±MET±TZD | 53.11±8.34 | 61.70 | NR | NR | NR | 12 | 8.4±0.795 | 12.78±6.58 | NR | 35.16±4.09 | Renal failure (Renal failure based on treatment emergent adverse events, vital signs, and laboratory measurements) | Publications |
| Yang 2016^31^ | 444 | Dapagliflozin | Placebo | MET | 53.8±9.29 | 54.28 | NR | NR | NR | 24 | 8.13±0.81 | 4.94±4.31 | NR | 26.10±3.32 | Events related to renal function (AE related to renal function based on prespecified AE item from MedDRA) | Publications |
| Ji 2014^32^ | 393 | Dapagliflozin | Placebo | drug-na¨ıve | 51.3±10.67 | 65.39 | NR | NR | NR | 24 | 8.26±0.89 | 1.38±2.40 | NR | 25.62 ±3.465 | Renal impairment (Renal impairment based on prespecified AE item from MedDRA) | Publications |
| Schumm-Draeger 2015^33^ | 300 | Dapagliflozin | Placebo | MET | 57.44±9.59 | 47.49 | NR | 65.22^a^ | NR | 16 | 7.81±0.78 | 5.37±4.15 | NR | 32.36±4.90 | Renal impairment/failure (Renal impairment/failure- based on prespecified AE item from MedDRA) | Publications |
| Matthaei 2015^34^ | 219 | Dapagliflozin | Placebo | MET+SU | 61.0±9.42 | 49.10 | NR | NR | 86.11 | 52 | 8.16±0.890 | 9.25±6.34 | 73.15 | 31.97±4.702 | Renal impairment/failure (renal impairment/failure (e.g. renal Cr clearance) based on prespecified AE items from MedDRA) | Publications |
| Mathieu 2015^35^ | 320 | Dapagliflozin | Placebo | SAXA+MET | 55.1±9.10 | 45.60 | 92.5±22.0^a^ | NR | NR | 52 | 8.2±0.97 | 7.6±6.1 | NR | 31.7±5.1 | GFR decrease | Publications (AEs of special interest Renal events) |
| Bolinder 2014^36^ | 182 | Dapagliflozin | Placebo | MET | 60.7±7.49 | 55.60 | NR | 61.11^a^ | 26.11 | 102 | 7.17±0.489 | 5.75±4.91 | NR | 31.87±3.882 | Renal impairment Renal impairment/failure based on prespecified AE items from MedDRA13.0) | Publications |
| Jabbour 2014^37^ | 451 | Dapagliflozin | Placebo | SIT±MET | 54.9±10.30 | 54.80 | NR | NR | NR | 48 | 7.93±0.792 | 5.67±5.14 | NR | NR | Renal impairment (Renal impairment (calculated creatinine clearance, estimated glomerular filtration rate)) | Publications (AEs of special interest Renal events) |
| Wilding 2014^38^ | 605 | Dapagliflozin | Placebo | INS ± OAD | 59.14±8.42 | 47.16 | NR | NR | NR | 104 | 8.56±0.83 | 13.59±7.48 | NR | 33.16±5.43 | Renal impairment/failure (Renal impairment/failure based on prespecified AE item from MedDRA and laboratory values) | Publications |
| Strojek 2014^39^ | 442 | Dapagliflozin | Placebo | GLI | 59.78±9.42 | 47.49 | NR | NR | 35.62 | 48 | 8.11±0.77 | 7.33±5.63 | NR | 29.77±5.14 | Renal impairment/ failure (Renal impairment/failure based on prespecified AE item from MedDRA and laboratory) | Publications |
| **DPP-4 Inhibitor vs. Control (22 studies)** | | | | | | | | | | | | | | |  |  |
| White 2013^40^ | 5380 | Alogliptin | Placebo | Standard care | 60.9±9.92 | 67.90 | 70.93±21.417^a^ | 84.41^a^ | 100^g^ | 164 | 8.03±1.09 | 9.16±8.159 | 81.99 | 29.48±5.591 | Renal failure, renal failure chronic and renal impairment | Based on prespecified SAE item from MedDRA 16.0 (Clinitrial trial registration) |
|  |  |  |  |  |  |  |  |  |  |  |  |  |  |  | Renal failure acute |  |
| Lewin 2012^41^ | 245 | Linagliptin | Placebo | SU | 56.9±9.9 | 52.70 | NR | 46.12 | NR | 18 | 8.61±0.81 | NR | NR | 28.33±5.04 | Renal failure acute | Based on prespecified SAE item from MedDRA 12.1 (Clinitrial trial registration) |
| NCT  02897349^42^ | 206 | Linagliptin | Placebo | INS±MET | 58.7±10.2 | 51.50 | NR | NR | NR | 25.1 | NR | NR | NR | NR | Acute kidney injury | Based on prespecified SAE item from MedDRA 21.1 (Clinitrial trial registration) |
| Yki-Järvinen 2013^43^ | 1263 | Linagliptin | Placebo | INS±MET±PIOG | 60.0±10.0 | 52.20 | NR | 56.23^a^ | NR | 52 | 8.30±0.85 | NR | NR | 31.0±5.16 | Renal failure chronic | Based on prespecified SAE item from MedDRA 14.0 (Clinitrial trial registration) |
|  |  |  |  |  |  |  |  |  |  |  |  |  |  |  | Renal failure acute |  |
| Ledesma 2019^44^ | 302 | Linagliptin | Placebo | INS±MET±ALP | 72.4±5.4 | 60.60 | NR | 85.76^a^ | NR | 24 | 8.2±0.8 | NR | NR | 28.09±5.68 | Renal impairment | Based on prespecified SAE item from MedDRA 20.0 (Clinitrial trial registration) |
|  |  |  |  |  |  |  |  |  |  |  |  |  |  |  | Acute kidney injury |  |
| MARLINA- T2D™  (Cooper) 2019^45^ | 360 | Linagliptin | Placebo | drug-na¨ıve/OAD±basal insulin | 60.6±9.6 | 63.61 | 98.65±46.77^b^ | 92.78^f^ | NR | 24 | 7.84±0.83 | NR | 100 | 28.45±4.85 | Acute kidney injury | Based on prespecified SAE item from MedDRA 18.1 (Clinitrial trial registration) |
| CARMELINA (Inagaki )  2019^46^ | 6991 | Linagliptin | Placebo | INS±MET±OAD | 65.9±9.1 | 62.90 | 54.6±25.0^a^ | 89.57^a^ | NR | 114.7 | 7.95±1.01 | 14.75±9.45 | 81.07 | 31.35±5.35 | Sustained ESRD, death due to  kidney failure, or sustained  decrease of ≥40% in eGFR  from baseline | Publications |
|  |  |  |  |  |  |  |  |  |  |  |  |  |  |  | Acute kidney injury | Based on prespecified SAE item from MedDRA 21.0 (Clinitrial trial registration) |
| McGill 2013^47^ | 133 | linagliptin | Placebo | INS±OAD | 64.4±10.3 | 60.20 | 23.57±6.75^a^ | 100^a^ | NR | 52 | 8.2±1.0 | NR | NR | 32.0±5.8 | Renal impairment | Based on prespecified SAE item from MedDRA (Clinitrial trial registration) |
|  |  |  |  |  |  |  |  |  |  |  |  |  |  |  | Acute renal failure |  |
| Owens 2011^48^ | 1058 | Linagliptin | Placebo | MET+SUL | 58.1±9.8 | 47.20 | NR | 39.62^a^ | NR | 24 | 8.14±0.81 | NR | NR | 28.33±4.72 | Renal impairment | Based on prespecified SAE item from MedDRA 12.0 (Clinitrial trial registration) |
| Matthews 2019^49^ | 2001 | Vildagliptin | Placebo | MET | 54.3±9.39 | 47.00 | NR | 56.18^a^ | NR | 302.4 | 6.7±0.46 | 6.4±7.55^h^ | NR | 31.1±4.75 | Renal failure/impairment | Based on prespecified SAE item from MedDRA 21.1 (Clinitrial trial registration) |
| Frias 2019^50^ | 458 | Sitagliptin | Placebo | MET | 55.5±10.4 | 40.00 | 115.7±34.81 | NR | NR | 22 | 8.7±0.9 | 6.35±5.95 | NR | 31.25±6.29 | Acute kidney injury | Based on prespecified SAE item from MedDRA 20.1 (Clinitrial trial registration) |
| Barzilai 2011^51^ | 206 | Sitagliptin | Placebo | OAD | 71.9±6.0 | 47.10 | 71±19.02^i^ | NR | NR | 24 | 7.8±0.7 | 7.10±7.38 | NR | 30.95±6.57 | Renal Failure Acute | Based on prespecified SAE item from MedDRA 10.1 (Clinitrial trial registration) |
| Shankar 2017^52^ | 467 | Sitagliptin | Placebo | INS±MET | 57.6±8.8 | 52.70 | NR | NR | NR | 26 | 8.75±0.90 | 11.15±5.41 | NR | 26.0±2.95 | Glomerular filtration rate decreased | Publications (AEs of special interest Renal events) |
| TECOS  (Green) 2015^53^ | 14671 | Sitagliptin | Placebo | INS±OAD | 65.5±8.0 | 70.70 | 74.9±21.1^a^ | NR | 100 | 156 | 7.2±0.5 | 11.6±8.1 | 78.8 | 30.2±5.6 | Renal failure | Publications (AEs of special interest Renal events) |
|  |  |  |  |  |  |  |  |  |  |  |  |  |  |  | Renal failure acute | Based on prespecified SAE item from MedDRA 17.0 (Clinitrial trial registration) |
| Gantz 2017^54^ | 4202 | Omarigliptin | Placebo | Standard care | 63.6±8.5 | 70.20 | 86.15±25.0 | NR | NR | 234 | 8.01±0.87 | 12.05±7.8 | NR | 31.3±5.55 | Renal failure | Based on prespecified SAE item from MedDRA 19.0 (Clinitrial trial registration) |
|  |  |  |  |  |  |  |  |  |  |  |  |  |  |  | Acute kidney injury |  |
| Chacra 2017^55^ | 213 | Omarigliptin | Placebo | INS±OAD | 65.2±9.6 | 61.50 | NR | 100^a^ | NR | 54 | 8.3±0.8 | 15.0±8.43 | NR | 30.1±5.78 | Worsening of renal function | Publications (AEs of special interest Renal events) |
| DeFronzo 2009^56^ | 562 | Saxagliptin | Placebo | MET | 54.70±9.94 | 50.18 | NR | NR | NR | 24 | 8.1±0.90 | 6.60±5.31 | NR | 31.50±4.88 | Renal failure acute | Based on prespecified SAE item from MedDRA 12.1 (Clinitrial trial registration) |
| Barnett 2013^57^ | 455 | Saxagliptin | Placebo | INS±MET | 57.2±9.37 | 41.30 | NR | NR | NR | 52 | 8.67±0.90 | 11.93±7.06 | NR | 32.33±5.36 | Renal impairment | Based on prespecified SAE item from MedDRA 13.1 (Clinitrial trial registration) |
|  |  |  |  |  |  |  |  |  |  |  |  |  |  |  | Renal failure acute |  |
| Müller- Wieland 2018^58^ | 626 | Saxagliptin | No treatment | MET | 58.30±8.69 | 62.62 | 87.6±19.49^a^ | NR | NR | 52 | 8.3±0.7 | 7.1±5.56 | 75.4 | 32.8±5.16 | Renal impairment/failure | Publications (AEs of special interest Renal events) |
| Dou 2018^59^ | 426 | Saxagliptin | Placebo | MET | 50.25±10.92 | 64.32 | NR | NR | NR | 24 | 9.4±1.05 | 0.85±1.84 | NR | 26.6±3.45 | Renal impairment | Publications (AEs of special interest Renal events) |
| Nowicki 2011^60^ | 170 | Saxagliptin | Placebo | INS±OAD | 66.5±8.66 | 42.90 | 30.95±13.35^i^ | 100^i^ | NR | 12 | 8.3±1.17 | 16.65±8.14 | NR | 30.7±6.46 | Renal Impairment (basedonCrCl values (LOCF analysis)) | Publications (AEs of special interest Renal events) |
| SAVOR-  TIMI53 (Mosenzon) 2017^61^ | 16492 | Saxagliptin | Placebo | INS±OAD | 65±8.55 | 66.90 | 71.70±4.58^a^ | 38.48**^f^** | 78.32 | 109.5 | 7.75±0.36 | 10.72±2.27 | 82.16 | 31.1±5.6 | Composite end point of death, doubling of serum creatinine levels or creatinine .6.0 mg/dL (530 mmol/L), initiation of chronic dialysis, and/or renal transplantation | Publications |
|  |  |  |  |  |  |  |  |  |  |  |  |  |  |  | Renal failure acute | Based on prespecified SAE item from MedDRA 15.1 (Clinitrial trial registration) |
| **GLP-1 agonist vs. Control (25 studies)** | | | | | | | | | | | | | | |  |  |
| Aroda 2016^62^ | 736 | Lixisenatide | No treatment | Insulin glargine±MET | 60.0±9.1 | 46.70 | NR | NR | NR | 30 | 8.08±0.71 | 12.08±6.74 | NR | 31.14±4.20 | Renal impairment | Based on prespecified SAE item from MedDRA 18.0 (Clinitrial trial registration) |
| Rosenstock 2014^63^ | 859 | Lixisenatide | Placebo | SU±MET | 57.2±9.9 | 50.5 | NR | NR | NR | 120 | 8.25±0.85 | 9.33±6.07 | NR | 30.20±6.60 | Renal failure acute | Based on prespecified SAE item from MedDRA 13.1 (Clinitrial trial registration) |
| Riddle 2013^64^ | 495 | Lixisenatide | Placebo | Basal INS±Met | 57.2±9.6 | 46.10 | NR | NR | NR | 125 | 8.40±0.87 | 12.46±6.80 | NR | 32.13±6.22 | Renal failure | Based on prespecified SAE item from MedDRA 13.1 (Clinitrial trial registration) |
| Meneilly 2017^65^ | 350 | Lixisenatide | Placebo | Basal INS±OAD | 74.2±3.9 | 52.00 | NR | NR | NR | 24 | 8.04±0.71 | 14.13±7.62 | 73.7 | 30.0±4.1 | Renal failure acute | Based on prespecified SAE item from MedDRA 17.1 (Clinitrial trial registration) |
| ELIXA (Muskiet) 2018^66^ | 6068 | Lixisenatide | Placebo | INS±OAD | 60.3±9.7 | 69.30 | 76.0±21.3^a^ | 76.21^a^ | 100 | 108.3 | 7.68±1.3 | 9.29±8.25 | 84.97 | 30.16±5.69 | Composite renal outcome (development of new-onset macroalbuminuria, decline in estimated glomerular filtration rate [or increase in creatinine], progression to end-stage kidney disease, or death attributable to kidney causes) | Publications |
|  |  |  |  |  |  |  |  |  |  |  |  |  |  |  | Renal failure acute | Based on prespecified SAE item from MedDRA 17.1 (Clinitrial trial registration) |
| Davies 2017^67^ | 279 | Oral Semaglutide | Placebo | drug-na¨ıve±met | 56.98±10.82 | 63.80 | NR | NR | NR | 31 | 7.85±0.71 | 5.85±4.90 | NR | 31.7±4.49 | Renal failure acute | Based on prespecified SAE item from MedDRA 20.0 (Clinitrial trial registration) |
| Zinman 2019^68^ | 731 | Oral Semaglutide | Placebo | INS±MET | 61.0±10.0 | 54.0 | 92.0±15.0^b^ | NR | NR | 52 | 8.2±0.7 | 15.0±8.1 | NR | 31.0±6.7 | Acute kidney injury | Based on prespecified SAE item from MedDRA 20.1 |
| Zinman 2019  (NCT03086330)^69^ | 302 | Semaglutide | Placebo | SGLT-2 inhibitor±SU or MET | 57.0±9.5 | 58.30 | 95.2±15.2^b^ | NR | NR | 30 | 8.0±0.8 | 9.7±6.1 | NR | 31.9±6.6 | Acute kidney injury | Based on prespecified SAE item from MedDRA 21.0 |
| Husain 2019^70^ | 3183 | Oral Semaglutide | Placebo | INS±OAD | 66.0±7.0 | 68.4 | 74.0±21.0^b^ | 70.5^b^ | 84.7^j^ | 82 | 8.2±1.6 | 14.9±8.5 | NR | 32.3±6.5 | Renal failure/ impairment | Based on prespecified SAE item from MedDRA 20.1 |
|  |  |  |  |  |  |  |  |  |  |  |  |  |  |  | Acute kidney injury |  |
| PIONEER 5  (Mosenzon)  2019^71^ | 324 | Oral Semaglutide | Placebo | Basal insulin±MET OR MET±SUL | 70.0±8.0 | 48.10 | 48.0±10.0^b^ | 100^b^ | NR | 31 | 8.0±0.7 | 14.0±8.0 | NR | 32.4±5.4 | Renal impairment | Based on prespecified SAE item from MedDRA 20.0 (Clinitrial trial registration) |
|  |  |  |  |  |  |  |  |  |  |  |  |  |  |  | Acute kidney injury | EAC-confirmed events |
| SUSTAIN-6  (Marso) 2016^72^ | 3297 | Semaglutide | Placebo | Standard-of-care treatment | 64.6±7.4 | 60.72 | 80(61-92)77.67(23.0) | 69.97^a^ | 83 | 104 | 8.7±1.46 | 13.9±8.11 | 83.5 | 32.8±6.2 | Composite renal outcome (new or worsening nephropathy includes persistent macroalbuminuria, persistent doubling of the serum creatinine level and a creatinine clearance of less than 45 ml per minute per 1.73 m2 of body-surface area (according to the Modification of Diet in Renal Disease criteria), or the need for continuous renal-replacement therapy)) | Publications |
|  |  |  |  |  |  |  |  |  |  |  |  |  |  |  | Acute renal failure |  |
| REWIND (Gerstein) 2019^73^ | 9901 | Dulaglutide | Placebo | Basal insulin±OAD | 66.2±6.5 | 53.65 | 76.9±22.75^a^ | 71.74^a^ | 31 | 282 | 7.35±1.1 | 10.55±7.25 | 81.49 | 32.3±5.75 | Composite renal outcome (development of new-onset macroalbuminuria, decline in estimated glomerular filtration rate [or increase in creatinine], progression to end-stage kidney disease, or death attributable to kidney causes) | Publications |
|  |  |  |  |  |  |  |  |  |  |  |  |  |  |  | Acute kidney injury |  |
| EXSCEL (Holman)  2017^74^ | 14752 | Exenatide（2mg once weekly ） | Placebo | standard-of-care treatment | 61.9±9.4 | 62.0 | 76.65(22.87)^a^ | 70.98^a^ | 88.83 | 166.9 | 8.1±1.0 | 13.1±8.3 | 71.91 | 32.7±64 | Composite renal outcome (development of new-onset macroalbuminuria, decline in estimated glomerular filtration rate [or increase in creatinine], progression to end-stage kidney disease, or death attributable to kidney causes) | Publications |
|  |  |  |  |  |  |  |  |  |  |  |  |  |  |  | Acute kidney injury | Based on prespecified SAE item from MedDRA 19.1 (Clinitrial trial registration) |
| DeFronzo 2010^75^ | 92 | Exenatide | No treatment | MET | 55.08±10.26 | 51.09 | NR | NR | NR | 20 | 7.85±0.11 | 4.7±3.7 | NR | 32.5±4.3 | Renal failure acute | Based on prespecified SAE item from MedDRA 11.0 (Clinitrial trial registration) |
| DURATION-7  (Guja) 2018^76^ | 464 | Exenatide (2mg once weekly ） | Placebo | Basal insulin glargine±MET | 57.7±9.67 | 47.8 | 90.9±18.5^b^ | NR | NR | 28 | 8.53±0.91 | 11.3±6.3 | NR | 33.7±6.4 | Acute kidney injury | Based on prespecified SAE item from MedDRA 19.0 (Clinitrial trial registration) |
| Reusch 2014^77^ | 310 | Albiglutide | Placebo | PIO±MET | 55.0±9.67 | 59.80 | NR | NR | NR | 156 | 8.1±0.9 | 8.0±5.8 | NR | 34.1±5.8 | Renal impairment | Based on prespecified SAE item from MedDRA (Clinitrial trial registration) |
| Harmony Outcomes (Hernandez) 2018^78^ | 9463 | Albiglutide | Placebo | standard-of-care treatment | 64.1±8.68 | 69.40 | 79.0±25.5 | NR | 100 | 83.4 | 8.74±1.5 | 14.15±8.75 | 67.74 | 32.3±5.9 | Renal impairment | Based on prespecified SAE item from MedDRA 21.0 (Clinitrial trial registration) |
|  |  |  |  |  |  |  |  |  |  |  |  |  |  |  | Acute kidney injury |  |
| NCT01733758^79^ | 387 | Albiglutide | Placebo | OAD | 58.40±9.71 | 75.19 | NR | NR | NR | 52 | NR | NR | NR | NR | Renal failure acute | Based on prespecified SAE item from MedDRA (Clinitrial trial registration) |
| NCT02964247^80^ | 303 | Liraglutide | Placebo | SGLT-2 inhibitor±MET | 55.15±10.02 | 60.40 | NR | NR | NR | 26 | NR | NR | NR | NR | Acute kidney injury | Based on prespecified SAE item from MedDRA 20.0 (Clinitrial trial registration) |
| Davies 2016^81^ | 279 | Liraglutide | Placebo | Ins±OAD | 67.2±8.2 | 50.50 | 45.45±0.24^a^ | 100^a^ | NR | 26 | 8.04±0.823 | 15.06±8.27 | NR | 33.94±5.42 | GFR rate decreased and renal impairment | Publications (vocabulary, Version 16.0) |
| Gough 2014^82^ | 1246 | Liraglutide | No treatment | Insulin degludec | 55.03±9.83 | 50.96 | NR | NR | NR | 52 | 8.3±0.9 | 6.73±5.17 | NR | 3.2±5.23 | Renal failure acute | Publications (vocabulary, Version 15.0) |
| Buse 2014^83^ | 413 | Liraglutide | No treatment | Insulin degludec | 57.2±9.7 | 54.80 | NR | NR | NR | 27 | 8.8±0.7 | 10.5±6.53 | NR | 33.7±6.0 | Renal failure acute | Based on vocabulary, Version 15.0 (Clinitrial trial registration) |
| Nauck 2009^84^ | 845 | Liraglutide | No treatment | MET | 56.57±9.67 | 58.46 | NR | NR | NR | 26 | 8.35±0.93 | 7.30±5.14 | NR | 30.94±4.69 | Renal failure acute | Based on prespecified SAE item from MedDRA 11.1 (Clinitrial trial registration) |
| PIONEER 4 (Pratley)  2019^85^ | 711 | Liraglutide/Oral Semaglutide | Placebo | MET±SGLT2i | 56.0±10.0 | 52.00 | 96.0±15.0^b^ | NR | NR | 52 | 8.0±0.7 | 7.6±5.5 | NR | 33.0±6.3 | Renal failure | Based on prespecified SAE item from MedDRA 20.1 (Clinitrial trial registration) |
| LEADER (Mann)  2017^86^ | 9340 | Liraglutide | placebo | drug-naive | 64.3±7.2 | 64.30 | 80.4^a^(mean) | 64.94^a^ | 81 | 197.6 | 8.69±1.51 | 12.85±7.98 | 82.88 | 32·5 ±6·3 | Composite renal outcome (development of new-onset macroalbuminuria, decline in estimated glomerular filtration rate [or increase in creatinine], progression to end-stage kidney disease, or death attributable to kidney causes) | Publications |
|  |  |  |  |  |  |  |  |  |  |  |  |  |  |  | Acute kidney injury | Based on prespecified SAE item from MedDRA (Clinitrial trial registration) |
| **SGLT-2 Inhibitor vs. DPP-4 Inhibitor (5 studies)** | | | | | | | | | | | | | | |  |  |
| DeFronzo 2018^87^ | 803 | Empagliflozin | Linagliptin | ±MET | 55.17±10.36 | 52.81 | 89.67±19.28^a^ | 53.3^a^ | NR | 52 | 8.02±0.92 | NR | NR | 31.3±5.6 | Renal failure acute | Based on prespecified SAE item from MedDRA 16.0 (Clinitrial trial registration) |
| Rosenstock 2015  (NCT01606007)^88^ | 355 | Dapagliflozin | Saxagliptin | MET | 54.05±9.65 | 51.55 | 93.21±19.69^a^ | NR | NR | 24 | 8.95±1.11 | 7.8±5.47 | NR | 31.63±5.23 | GFR decrease | Publications (AEs of special interest Renal events) |
| Lavalle-  González 2013^89^ | 1101 | Canagliflozin | Sitagliptin | MET | 55.43±9.38 | 46.41 | NR | NR | NR | 52 | 7.9±0.9 | 6.87±5.33 | NR | 31.93±6.28 | Renal failure acute | Based on prespecified SAE item from MedDRA 15.0 (Clinitrial trial registration) |
| Pratley 2018^90^ | 497 | Ertugliflozin | Sitagliptin | MET | 54.95±10.39 | 56.54 | 92.25±19.43^a^ | NR | NR | 54 | 8.55±1.0 | NR | NR | 31.75±6.34 | Acute kidney injury | Based on prespecified SAE item from MedDRA 19.0 (Clinitrial trial registration) |
| Scott 2018^91^ | 613 | Dapagliflozin | Sitagliptin | MET+SU | 67.1±8.5 | 57.90 | 78.15±11.87^b^ | 100^b^ | NR | 24 | 7.7±0.7 | 10.60±7.20 | NR | 31.65±5.50 | eGFR decrease from baseline >30% | Publications (AEs of special interest Renal events) |
| **GLP-1 Agonist vs. DPP-4 Inhibitor (6 studies)** | | | | | | | | | | | | | | |  |  |
| Rosenstock 2019^92^ | 1864 | Oral Semaglutide | Sitagliptin | MET±SUL | 58.0±10.0 | 52.80 | 95.75±15.50^b^ | NR | NR | 78 | 8.3±0.9 | 8.55±6.0 | NR | 32.5±6.4 | Acute kidney injury | Based on prespecified SAE item from MedDRA 20.1 (Clinitrial trial registration) |
| Ahrén 2017^93^ | 1231 | Semaglutide | Sitagliptin | MET±TZD | 55.1±10.0 | 50.60 | 119.52±34.92^a^ | NR | NR | 56 | 8.07±0.93 | 6.57±5.14 | 53.39 | 32.47±6.2 | Renal failure | Based on prespecified SAE item from MedDRA (Clinitrial trial registration) |
| Pieber 2019^95^ | 504 | Oral Semaglutide | Sitagliptin | OAD | 57.0±10.0 | 56.50 | 96.15±15.02^b^ | NR | NR | 52 | 8.3±0.6 | 8.8±6.25 | NR | 31.5±6.3 | Acute kidney injury | EAC-confirmed events |
| Leiter 2014 (NCT01098539)^96^ | 495 | Albiglutide | Sitagliptin | OAD | 63.3±8.69 | 53.70 | NR | 100^a^ | NR | 60 | 8.13±1.036 | 10.83±7.403 | NR | 30.39±5.644 | Renal failure/ impairment | Based on prespecified SAE item from MedDRA (Clinitrial trial registration) |
| Pratley 2011^97^ | 665 | Liraglutide | Sitagliptin | MET | 55.3±9.2 | 52.90 | NR | NR | NR | 78 | 8.4±0.8 | 6.2±5.1 | NR | 32.8±5.2 | Renal failure acute | Based on prespecified SAE item from MedDRA (Clinitrial trial registration) |
| NCT 00976937^98^ | 319 | Lixisenatide | Sitagliptin | MET | 43.1±4.9 | 40.10 | NR | NR | NR | 24 | 8.12±0.93 | 4.42±3.70 | NR | 36.76±6.80 | Renal failure | Based on prespecified SAE item from MedDRA 13.1 (Clinitrial trial registration) |
| **GLP-1 Agonist vs. SGLT-2 Inhibitor (1 studies)** | | | | | | | | | | | | | | | | |
| Rodbard 2019^94^ | 822 | Oral Semaglutide | Empagliflozin | MET | 58.0±10.0 | 50.50 | 95.0±15.0^b^ | NR | NR | 52 | 8.1±0.9 | 7.4±6.1 | NR | 32.8±6.1 | Acute kidney injury | Incidence Of EAC-Confirmed Events |

Mean data reported as mean ± standard deviation unless otherwise specified.

^a^ measured using the Modification of Diet in Renal Disease Study(MDRD) formula; ^b^ calculated using the Chronic Kidney Disease Epidemiology Collaboration (CKD-EPI) formula; ^c^ (AKI) was addressed through a standard monitoring protocol using creatinine-based KDIGO definitions; ^d^ Medical history of CV disease or heart failure; ^e^ documented pre-existing cardiovascular disease (CVD), and a history of hypertension; ^f^ UACR ≥30mg/g; ^g^ Cardiovascular risk factors and history; ^h^ the unit is months; ^i^ Creatinine clearance estimated via Cockcroft-Gault, mL/min; ^j^ Age ≥50 yr and established CVD or chronic kidney disease.

NR, not reported; BMI, body mass index; SGLT-2, sodium-glucose cotransporter-2; GLP-1, glucagon-like peptide- 1; DPP-4, dipeptidyl peptidase-4; HbA_1c_, glycated haemoglobin; CKD, chronic kidney disease; eGFR, estimated glomerular filtration rate; SU, sulfonylureas; OAD, oral antidiabetic drugs; INS, insulin; PIOG, pioglitazone; SAXA, saxagliptin; LINA, linagliptin; MET, metformin; PLA, placebo; TZD, thiazolidinediones; CVD, cardiovascular disease; GLI, glimepiride; ACEi/ARBs, angiotensin converting enzyme inhibitors/angiotensin receptor blockers; ALP,alpha-glucosidase inhibitors.

**Appendix 3**

**Risk of bias assessment**

**Supplemental Table 3**. Risk of bias of individual trials

| Trial | Sequence  generation | Allocation  concealment | Blinding | Detection  Bias | Attrition  Bias |
| --- | --- | --- | --- | --- | --- |
| Stenl ¨of 2013^1^ | Unclear | Unclear | Low | Low | Low |
| Yale 2014^2^ | Low | Unclear | Low | Low | Low |
| Bode 2015^3^ | Low | Unclear | Low | Low | High |
| CREDENCE (Mahaffey) 2019^4^ | Low | Low | Low | Low | Low |
| CANVAS (Perkovic) 2018^5^ | Low | Low | Low | Low | Low |
| Rosenstock 2016^6^ | Low | Low | Low | Low | Low |
| Qiu 2014^7^ | Low | Unclear | Low | Low | Low |
| Allegretti 2019^8^ | Low | Low | Low | Low | Low |
| VERTIS RENAL (Grunberger) 2018^9^ | Low | Unclear | Low | Low | Low |
| Haneda 2016^10^ | Unclear | Unclear | Low | Low | Low |
| EMPA-REG OUTCOME  (Monteiro) 2019^11^ | Low | Low | Low | Low | Low |
| Barnett 2014^12^ | Low | Low | Low | Low | Low |
| Rosenstock 2015^13^ | Low | Unclear | Low | Low | Low |
| Rosenstock 2014^14^ | Low | Unclear | Low | Low | Low |
| Häring 2014^15^ | Low | Unclear | Low | Low | High |
| Haering 2015^16^ | Low | Unclear | Low | Low | High |
| Roden 2015^17^ | Low | Low | Low | Low | High |
| Søfteland 2017^18^ | Low | Unclear | Low | Low | Low |
| Kohan 2014^19^ | Unclear | Unclear | Low | Low | High |
| Cefalu 2015^20^ | Unclear | Unclear | Low | Low | Low |
| Leiter 2014^21^ | Unclear | Unclear | Low | Low | Low |
| Kaku 2014^22^ | Unclear | Unclear | Low | Low | Low |
| DECLARE–TIMI 58 (mosenzon) 2019^23^ | Low | Low | Low | Low | Low |
| Pollock 2019^24^ | Low | Unclear | Low | Low | Low |
| Heerspink 2016^25^ | Low | Unclear | Low | Low | Unclear |
| Bailey 2013^26^ | Low | Low | Low | Low | Low |
| Bailey 2012^27^ | Low | Unclear | Low | Low | Low |
| Bailey 2015^28^ | Low | Low | Low | Low | High |
| Araki 2016^29^ | Unclear | Unclear | Low | Low | Low |
| Wilding 2009^30^ | Unclear | Unclear | Low | Low | Low |
| Yang 2016^31^ | Low | Unclear | Low | Low | Low |
| Ji 2014^32^ | Low | Unclear | Low | Low | Low |
| Schumm-Draeger 2015^33^ | Low | Unclear | Low | Low | Low |
| Matthaei 2015^34^ | Low | Unclear | Low | Low | Low |
| Mathieu 2015^35^ | Low | Unclear | Low | Low | Low |
| Bolinder 2014^36^ | Low | Low | Low | Low | Low |
| Jabbour 2014^37^ | Unclear | Unclear | Low | Low | Low |
| Wilding 2014^38^ | Low | Low | Low | Low | Low |
| Strojek 2014^39^ | Low | Unclear | Low | Low | Low |
| White 2013^40^ | Low | Low | Low | Low | Low |
| Lewin 2012^41^ | Low | Low | Low | Low | Low |
| NCT  02897349^42^ | Unclear | Unclear | Low | Low | Low |
| Yki-Järvinen 2013^43^ | Low | Low | Low | Low | Low |
| Ledesma 2019^44^ | Low | Low | Low | Low | Low |
| MARLINA- T2D™  (Cooper) 2019^45^ | Low | Low | Low | Low | Low |
| CARMELINA (Inagaki )  2019^46^ | Low | Low | Low | Low | Low |
| McGill 2013^47^ | Unclear | Unclear | Low | Low | Low |
| Owens 2011^48^ | Unclear | Unclear | Low | Low | Low |
| Matthews 2019^49^ | Low | Low | Low | Low | Low |
| Frias 2019^50^ | Low | Low | Low | Low | Low |
| Barzilai 2011^51^ | Low | Low | Low | Low | Low |
| Shankar 2017^52^ | Unclear | Unclear | Low | Low | Low |
| TECOS  (Green) 2015^53^ | Low | Low | Low | Low | Low |
| Gantz 2017^54^ | Low | Low | Low | Low | Low |
| Chacra 2017^55^ | Low | Low | Low | Low | Low |
| DeFronzo 2009^56^ | Low | Low | Low | Low | High |
| Barnett 2013^57^ | Low | Low | Low | Low | Low |
| Müller- Wieland 2018^58^ | Low | Low | Low | Low | Low |
| Dou 2018^59^ | Low | Low | Low | Low | Low |
| Nowicki 2011^60^ | Low | Low | Low | Low | High |
| SAVOR-  TIMI53 (Mosenzon) 2017^61^ | Low | Low | Low | Low | Low |
| Aroda 2016^62^ | Low | Low | High | High | Low |
| Rosenstock 2014^63^ | Unclear | Unclear | Low | Low | High |
| Riddle 2013^64^ | Low | Low | Low | Low | Low |
| Meneilly 2017^65^ | Low | Low | Low | Low | Low |
| ELIXA (Muskiet) 2018^66^ | Low | Low | Low | Low | Low |
| Davies 2017^67^ | Low | Low | High | High | Low |
| Zinman 2019^68^ | Low | Low | Low | Low | Low |
| Zinman 2019  (NCT03086330)^69^ | Low | Low | Low | Low | Low |
| Husain 2019^70^ | Low | Low | Low | Low | Low |
| PIONEER 5  (Mosenzon)  2019^71^ | Low | Low | Low | Low | Low |
| SUSTAIN-6  (Marso) 2016^72^ | Low | Low | Low | Low | Low |
| REWIND (Gerstein) 2019^73^ | Low | Low | Low | Low | Low |
| EXSCEL (Holman)  2017^74^ | Low | Low | Low | Low | Low |
| DeFronzo 2010^75^ | Low | Unclear | Low | Low | Low |
| DURATION-7  (Guja) 2018^76^ | Low | Low | Low | Low | Low |
| Reusch 2014^77^ | Low | Low | Low | Low | High |
| Harmony Outcomes (Hernandez) 2018^78^ | Low | Low | Low | Low | Low |
| NCT01733758^79^ | Unclear | Unclear | Low | Low | Low |
| NCT02964247^80^ | Unclear | Unclear | Low | Low | Low |
| Davies 2016^81^ | Low | Low | Low | Low | High |
| Gough 2014^82^ | Low | Low | High | High | Low |
| Buse 2014^83^ | Low | Low | Low | Low | Low |
| Nauck 2009^84^ | Low | Low | Low | Low | High |
| PIONEER 4 (Pratley)  2019^85^ | Low | Low | Low | Low | Low |
| LEADER (Mann)  2017^86^ | Low | Low | Low | Low | Low |
| DeFronzo 2018^87^ | Low | Unclear | Low | Low | Low |
| Rosenstock 2015  (NCT01606007)^88^ | Low | Unclear | Low | Low | High |
| Lavalle-  González 2013^89^ | Low | Low | Low | Low | High |
| Pratley 2018^90^ | Low | Low | Low | Low | Low |
| Scott 2018^91^ | Low | Low | Low | Low | Low |
| Rosenstock 2019^92^ | Low | Low | Low | Low | Low |
| Ahrén 2017^93^ | Low | Low | Low | Low | Low |
| Rodbard 2019^94^ | Low | Low | High | High | Low |
| Pieber 2019^95^ | Low | Low | High | High | Low |
| Leiter 2014 (NCT01098539)^96^ | Low | Low | Low | Low | High |
| Pratley 2011^97^ | Low | Low | High | High | High |
| NCT 00976937^98^ | Unclear | Unclear | Low | Low | Low |

**Supplemental Figure 1**. Risk of bias assessment for overall included studies


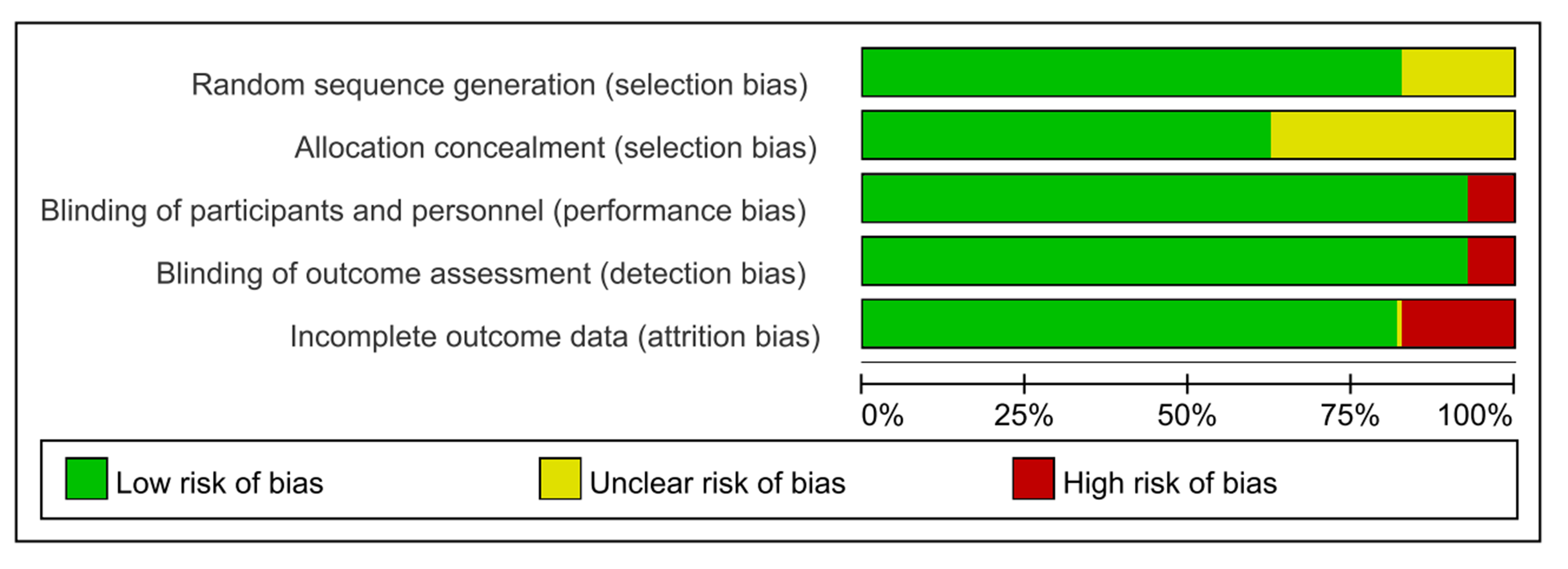


Total of 98 trials were assessed for risk of bias

**Supplemental Table 4A.** Contribution summary of risk of bias assessments for any direct comparisons included in the network meta-analysis on composite renal events.

| **Direct comparison** | **Risk of bias assessment** |
| --- | --- |
| SGLT-2i vs Control | Low |
| SGLT-2i vs DPP-4i | Low |
| Control vs DPP-4i | Low |
| Control vs GLP-1a | Low |
| DPP-4i vs GLP-1a | Low |

**Supplemental Table 4B.** Contribution summary of risk of bias assessments for any direct comparisons included in the network meta-analysis on acute kidney injury events.

| **Direct comparison** | **Risk of bias assessment** |
| --- | --- |
| SGLT-2i vs Control | Low |
| SGLT-2i vs DPP-4i | Moderate |
| SGLT-2i vs GLP-1a | High |
| Control vs DPP-4i | Low |
| Control vs GLP-1a | Low |
| DPP-4i vs GLP-1a | High |

Abbreviation: Control represents either placebo or no treatment; DPP-4, dipeptidyl

peptidase 4; GLP-1, glucagon-like peptide 1; and SGLT-2, sodium-glucose cotransporter 2.

**Supplemental Figure 2A.** Contribution plot for composite renal events


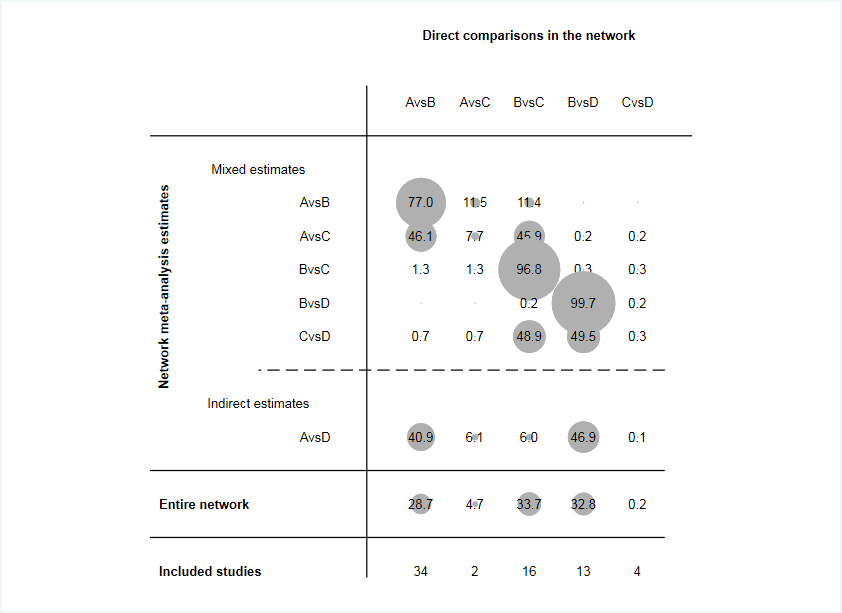


**Supplemental Figure 2B.** Contribution plot for acute kidney injury events


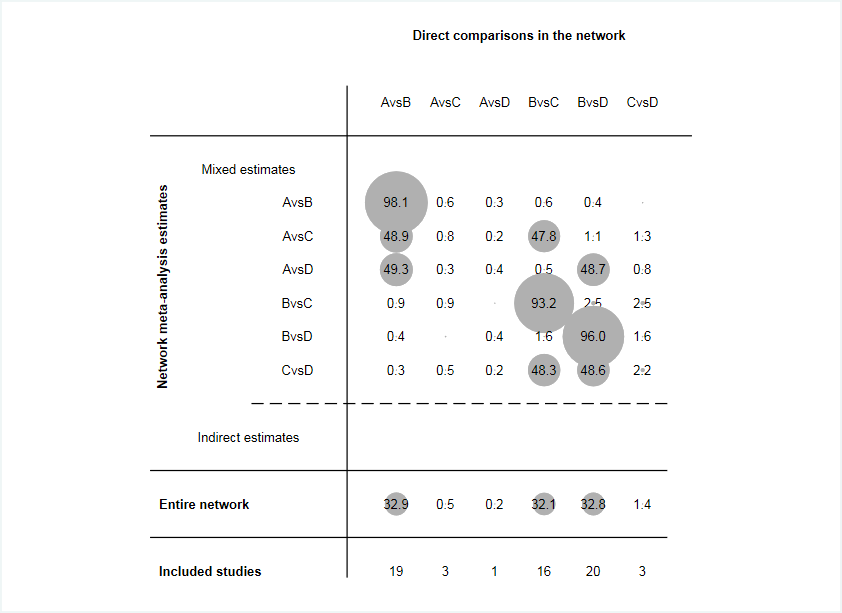


The size of the circle is proportional to the percentage contribution of the column-defining direct comparison to the row-defining network estimate.

Abbreviation: A, sodium-glucose cotransporter 2; B, placebo or no treatment; C, dipeptidyl peptidase 4 and D, glucagon-like peptide 1.

**Supplemental Table 5A.** The contribution of direct comparisons to mixed or indirect comparisons by risk of bias classification on composite renal events

| **Comparison** | **Risk of bias assessment** | | |
| --- | --- | --- | --- |
|  | **Low (%)** | **Moderate (%)** | **High (%)** |
| SGLT-2i vs Control | 100.0 | 0.0 | 0.0 |
| SGLT-2i vs DPP-4i | 100.0 | 0.0 | 0.0 |
| Control vs DPP-4i | 100.0 | 0.0 | 0.0 |
| Control vs GLP-1a | 100.0 | 0.0 | 0.0 |
| DPP-4i vs GLP-1a | 100.0 | 0.0 | 0.0 |
| SGLT-2i vs GLP-1a | 100.0 | 0.0 | 0.0 |
| Entire network | 100.0 | 0.0 | 0.0 |

**Supplemental Table 5B.** The contribution of direct comparisons to mixed or indirect comparisons by risk of bias classification on acute kidney injury events.

| **Comparison** | **Risk of bias assessment** | | |
| --- | --- | --- | --- |
|  | **Low (%)** | **Moderate (%)** | **High (%)** |
| SGLT-2i vs Control | 99.1 | 0.6 | 0.3 |
| SGLT-2i vs DPP-4i | 97.8 | 0.8 | 1.4 |
| SGLT-2i vs GLP-1a | 98.5 | 0.3 | 1.2 |
| Control vs DPP-4i | 96.6 | 0.9 | 2.5 |
| Control vs GLP-1a | 98.0 | 0.0 | 2.0 |
| DPP-4i vs GLP-1a | 97.2 | 0.5 | 2.3 |
| Entire network | 97.8 | 0.5 | 1.7 |

Abbreviation: Control represents either placebo or no treatment; DPP-4, dipeptidyl

peptidase 4; GLP-1, glucagon-like peptide 1; and SGLT-2, sodium-glucose cotransporter 2.

**Supplemental Figure 3A.** Contribution of summary RoB comparisons to each network estimate on composite renal events


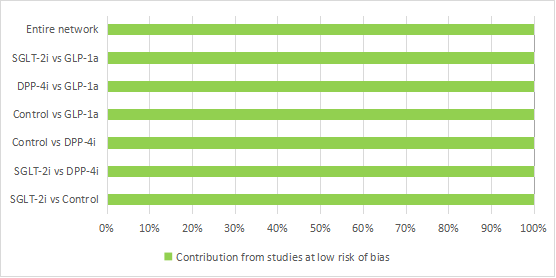


**Supplemental Figure 3B.** Contribution of summary RoB comparisons to each network estimate on acute kidney injury events


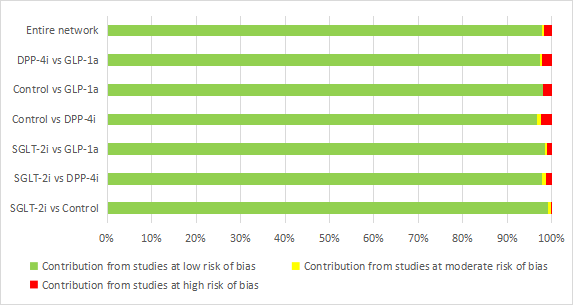


Based on the above assessment of RoB for each comparison and the contribution matrix detailing contribution of each direct comparison to all network estimates, the bar graphs above show the percentage of low, moderate and high RoB contributions for each network estimate.

The judgements about study limitations in each direct comparison is shown at the beginning of the graph. Each bar corresponds to a NMA relative treatment effect and shows how much information comes from comparisons at low risk of bias [green], moderate risk of bias [yellow] and high risk of bias [red].

**Supplemental Figure 4**. Comparison-adjusted funnel plot for the network meta-analysis of the three classes of glucose-lowering agents on composite kidney outcome (A) and acute kidney injury (B)


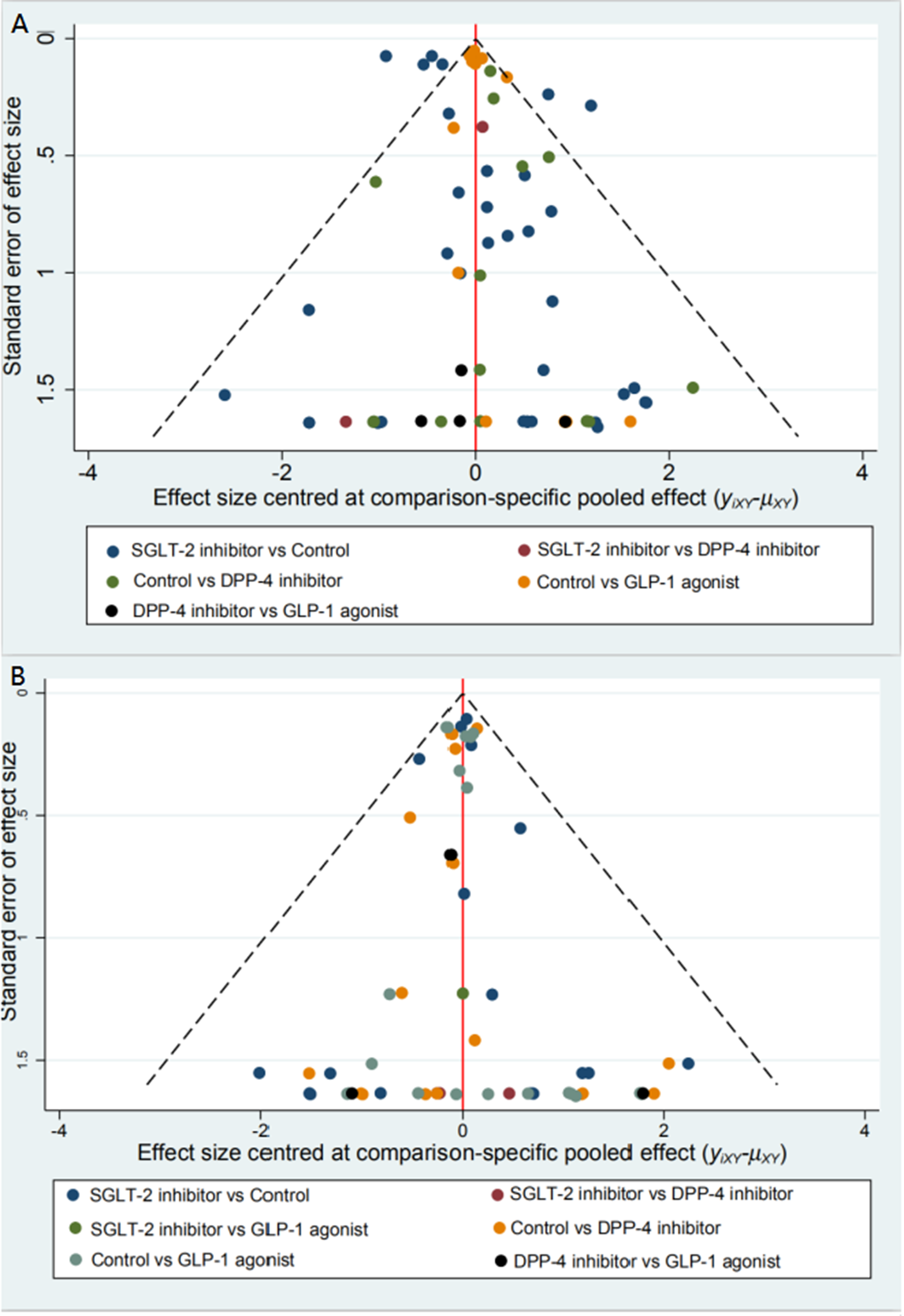


In this presentation, all studies are centered on the summary effect estimate of their respective comparisons [μXY (logOR for present study)] which is represented by the vertical red line. Individual study-level effect size is represented by yiXY [where X and Y are two study agents]. μ_xy_ is the comparison specific summary estimate for x versus y. Outer dotted lines represent a 95% CI for the difference between study-specific effect sizes and comparison-specific summary estimates. Please note that this is drawn only for comparisons with 2 or more studies.

**Appendix 4**

**Supplemental Table 6**. Pairwise meta-analysis of the effects of glucose-lowering drugs on the risk of composite renal events and acute kidney injury events

| **Item** | **Comparison** | **No**  **of studies** | **Intervention**  **(events/**  **patients)** | **Control (events/**  **patients)** | **Odds ratio**  **(95%CI)** | **Heterogeneity (I^2^ %)** | **Publication bias (Egger test, P-value)** |
| --- | --- | --- | --- | --- | --- | --- | --- |
| Composite renal events | SGLT-2 inhibitors vs. Control | 34 | 1325/26723 | 1506/20797 | 0.88 (0.68-1.14) | 70.1 | **0.004** |
|  | GLP-1 agonists  vs. Control | 13 | 1944/28183 | 2277/27571 | **0.84 (0.78-0.89)** | 0.0 | 0.128 |
|  | DPP-4 inhibitors  vs. Control | 16 | 1063/27795 | 1012/27023 | 1.05 (0.96-1.15) | 0.0 | 0.307 |
|  | GLP-1 agonists  vs. DPP-4 inhibitors | 4 | 3/2620 | 2/1280 | 0.87 (0.21-3.65) | 0.0 | - |
|  | SGLT-2 inhibitors vs. DPP-4 inhibitors | 2 | 17/485 | 14/482 | 1.23 (0.61-2.51) | 0.0 | - |
| Acute kidney injury events | SGLT-2 inhibitors vs. Control | 19 | 427/26040 | 385/20575 | **0.75 (0.65-0.86)** | 0.0 | 0.819 |
|  | GLP-1 agonists  vs Control | 20 | 351/32303 | 355/30192 | 0.97 (0.84-1.12) | 0.0 | 0.563 |
|  | DPP-4 inhibitors  vs. Control | 16 | 245/26395 | 218/25873 | 1.10 (0.92-1.32) | 0.0 | 0.961 |
|  | GLP-1 agonists  vs. DPP-4 inhibitors | 3 | 11/2087 | 4/935 | 0.93 (0.32-2.70) | 0.0 | - |
|  | SGLT-2 inhibitors vs. DPP-4 inhibitors | 4 | 0/1286 | 2/633 | 0.21 (0.03-1.31) | 0.0 | - |
|  | SGLT-2 inhibitors vs. GLP-1 agonists | 1 | 1/409 | 2/410 | 0.60 (0.08-4.56) | - | - |

CI, confidence interval.

Abbreviation: Control represents either placebo or no treatment; DPP-4, dipeptidyl peptidase 4; GLP-1, glucagon-like peptide 1; and SGLT-2, sodium-glucose cotransporter 2.

**Appendix 5**

**Deviance information criterion for model selection**

**Supplemental Table 7**. Deviance information criterion for model selection (fixed- vs. random-effects)

| Outcome | Model | Deviance Information  Criterion (DIC) | Accepted Model |
| --- | --- | --- | --- |
| Composite renal events | Fixed | 352.1 | Random |
|  | Random | 250.1 |  |
| Acute kidney injury events | Fixed | 216.0 | Random |
|  | Random | 217.5 |  |

**Appendix 6**

**Assessment of transitivity**

**Supplemental Figure 5A**. Box plots among trials included for composite renal events.


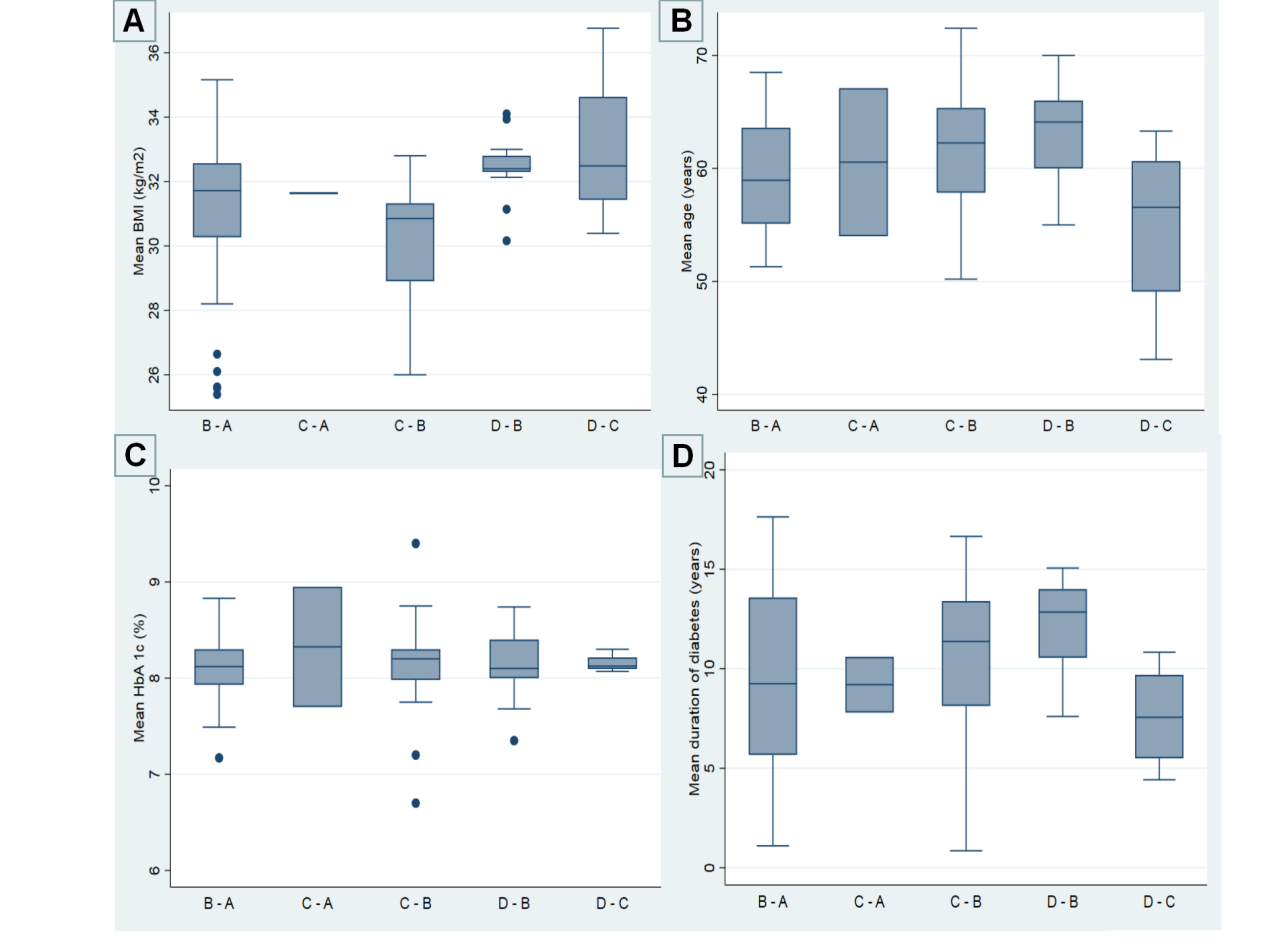


**Supplemental Figure 5B**. Box plots among trials included for acute kidney injury events.


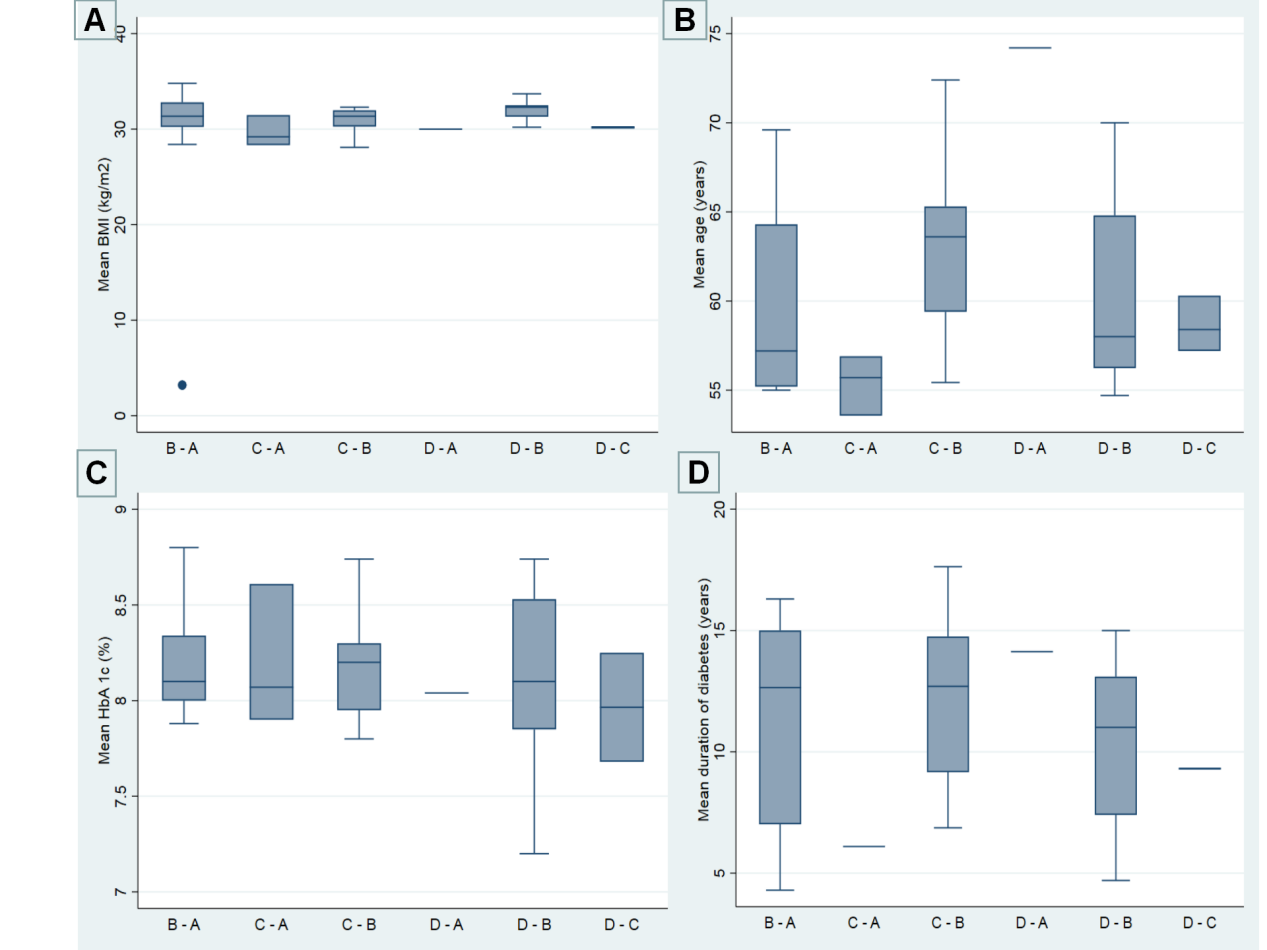


A. Mean BMI at baseline (kg/m^2^); B. Mean age at baseline (years); C. Mean baseline HbA1c (%); D. Mean baseline duration of type 2 diabetes (years). The x-coordinate is the comparison group. A, SGLT-2 inhibitor; B, Control; C, DPP-4 inhibitor; D, GLP-1 agonist.

Abbreviation: BMI, body mass index, calculated as weight in kilograms divided by height in meters squared; HbA1c, hemoglobin A1c; Control represents either placebo or no treatment; DPP-4, dipeptidyl peptidase 4; GLP-1, glucagon-like peptide 1; and SGLT-2, sodium-glucose cotransporter 2.

**Appendix 7**

**Evaluation of inconsistency**

**Supplemental Table 8A. Node-splitting meta-analysis of two comparisons for composite renal events**

| Comparison | Direct Effect | Indirect Effect | Network | P-value |
| --- | --- | --- | --- | --- |
| Control vs SGLT-2i | 0.10 (-0.23, 0.37) | -0.05 (-1.20, 1.20) | 0.09 (-0.22, 0.34) | 0.817 |
| DPP-4i vs SGLT-2i | -0.10 (-1.20, 1.10) | 0.01 (-0.50, 0.47) | -0.00 (-0.45, 0.42) | 0.867 |
| DPP-4i vs Control | -0.06 (-0.46, 0.31) | -0.24 (-1.20, 0.75) | -0.08 (-0.45, 0.27) | 0.743 |
| GLP-1a vs Control | -0.24 (-0.60, 0.10) | 0.16 (-1.70, 2.60) | -0.24 (-0.60, 0.10) | 0.472 |
| GLP-1a vs DPP-4i | 0.11 (-1.70, 2.20) | -0.17 (-0.69, 0.34) | -0.15 (-0.66, 0.34) | 0.786 |

**Supplemental Table 8B.** Node-splitting meta-analysis of two comparisons for acute kidney injury events

| Comparison | Direct Effect | Indirect Effect | Network | P-value |
| --- | --- | --- | --- | --- |
| Control vs SGLT-2i | 0.28 (0.11, 0.45) | 2.4 (0.76, 5.7) | 0.30 (0.13, 0.49) | **0.000** |
| DPP-4i vs SGLT-2i | 3.80 (0.74, 11.0) | 0.40 (0.13, 0.67) | 0.44 (0.20, 0.72) | **0.013** |
| GLP-1a vs SGLT-2i | 0.77 (-2.40, 2.80) | 0.25 (0.016, 0.53) | 0.28 (0.05, 0.50) | 0.681 |
| DPP-4i vs Control | 0.10 (-0.13, 0.33) | 0.40 (-0.56, 1.3) | 0.14 (-0.05, 0.32) | 0.612 |
| GLP-1a vs Control | -0.02 (-0.19, 0.14) | 0.09 (-0.65, 1.3) | -0.03 (-0.20, 0.13) | 0.733 |
| GLP-1a vs DPP-4i | 0.34 (-0.55, 2.1) | -0.17 (-0.44, 0.099) | -0.17 (-0.40, 0.11) | 0.335 |

Bold cells are significant inconsistency.

Abbreviation: Control represents either placebo or no treatment; DPP-4, dipeptidyl

peptidase 4; GLP-1, glucagon-like peptide 1; and SGLT-2, sodium-glucose cotransporter 2.

**Supplement 1.** Global inconsistency assesment

Design-by-treatment test for composite renal events

chi2( 2) = 0.21

Prob > chi2 = 0.9020

Design-by-treatment test for acute kidney injury events

chi2( 3) = 1.75

Prob > chi2 = 0.6259

**Appendix 8**

**Subgroup network meta-analyses**

**Supplemental Table 9A.** Subgroup network meta-analyses for composite renal events

| **Group/Subgroup** | **No**  **of studies** | **Events/**  **Patients** | **SGLT-2i vs Control** | **SGLT-2i vs DPP-4i** | **SGLT-2i vs GLP-1a** | **Control vs DPP-4i** | **Control vs GLP-1a** | **DPP-4i vs GLP-1a** | **Heterogeneity (*I* ^2^ %)** |
| --- | --- | --- | --- | --- | --- | --- | --- | --- | --- |
| Overall | 69 | 9163/ 162,959 | 0.92  (0.70, 1.24) | 1.0  (0.66, 1.59) | 1.09  (0.70, 1.56) | 1.26  (0.90, 1.80) | 1.15  (0.72, 1.89) | 1.16  (0.76, 1.88) | 10% |
| CVD | 17 | 6724/ 107,293 | 0.86  (0.56, 1.46) | 0.87  (0.43, 1.92) | 1.08  (0.56, 2.23) | 1.01  (0.58, 1.79) | 1.26  (0.77, 2.04) | 1.24  (0.58, 2.59) | 8% |
| Non-CVD | 52 | 2439/ 55,666 | 0.97  (0.65, 1.53) | 1.16  (0.62, 2.30) | 1.24  (0.61, 3.20) | 1.2  (0.66, 2.09) | 1.29  (0.69, 2.82) | 1.08  (0.49, 2.75) | 12% |
| CKD | 40 | 8932/ 144,670 | **0.57**  **(0.48, 0.74)** | **0.6**  **(0.46, 0.85)** | **0.7**  **(0.55, 0.97)** | 1.04  (0.84, 1.31) | **1.22**  **(1.02, 1.47)** | 1.18  (0.88, 1.55) | 13% |
| Non-CKD | 29 | 231/  18,298 | 1.74  (0.98, 2.93) | **8.23**  **(1.51, 58.48)** | **8.35**  **(1.12, 81.15)** | 4.73  (0.94, 32.97) | 4.89  (0.7, 44.35) | 1.05  (0.11, 9.04) | 13% |
| ACEi/ARB treatment | 21 | 8483/ 123,095 | 0.82  (0.56, 1.27) | 0.89  (0.48, 1.79) | 1.04  (0.57, 2.02) | 1.08  (0.66, 1.81) | 1.27  (0.79, 2.06) | 1.17  (0.58, 2.38) | 9% |
| Non-ACEi/ARB treatment | 48 | 680/  39,864 | 1.14  (0.76, 1.76) | 1.2  (0.66, 2.22) | 1.35  (0.76, 2.7) | 1.04  (0.61, 1.85) | 1.18  (0.76, 2.07) | 1.14  (0.58, 2.37) | 14% |
| Cardiovascular outcome | 16 | 8703/  133,294 | **0.5**  **(0.42, 0.62)** | **0.5**  **(0.38, 0.68)** | **0.62**  **(0.49, 0.81)** | 0.98  (0.81, 1.23) | **1.22**  **(1.05, 1.44)** | 1.24  (0.95, 1.61) | 1% |
| Non-cardiovascular outcome | 53 | 460/  29,665 | 1.33  (0.89, 1.92) | 1.49  (0.76, 2.91) | 1.84  (0.71, 5.07) | 1.12  (0.62, 2.08) | 1.39  (0.58, 3.59) | 1.24  (0.46, 3.56) | 14% |

**Supplemental Table 9B.** Subgroup network meta-analyses for AKI events

| **Group/Subgroup** | **No**  **of studies** | **Events/**  **Patients** | **SGLT-2i vs Control** | **SGLT-2i vs DPP-4i** | **SGLT-2i vs GLP-1a** | **Control vs DPP-4i** | **Control vs GLP-1a** | **DPP-4i vs GLP-1a** | **Heterogeneity (*I* ^2^ %)** |
| --- | --- | --- | --- | --- | --- | --- | --- | --- | --- |
| Overall | 61 | 2002/ 167,584 | **0.74**  **(0.62, 0.87)** | **0.67**  **(0.50, 0.86)** | **0.76**  **(0.59, 0.96)** | 0.90  (0.72, 1.10) | 1.00  (0.86, 1.20) | 1.10  (0.88, 1.50) | 15% |
| CVD | 16 | 1257/ 102, 969 | **0.73**  **(0.52, 0.99)** | **0.6**  **(0.36, 0.96)** | 0.74  (0.5, 1.11) | 0.82  (0.57, 1.17) | 1.02  (0.81, 1.32) | 1.24  (0.82, 1.97) | 8% |
| Non-CVD | 46 | 745/ 64,615 | 0.74  (0.48, 1.02) | 0.69  (0.33, 1.08) | 0.72  (0.33, 1.22) | 0.92  (0.56, 1.32) | 0.98  (0.55, 1.5) | 1.07  (0.59, 1.89) | 19% |
| CKD | 28 | 1737/ 139,306 | **0.73**  **(0.61, 0.87)** | **0.66**  **(0.49, 0.86)** | 0.8  (0.6, 1.05) | 0.9  (0.72, 1.1) | 1.09  (0.89, 1.33) | 1.22  (0.91, 1.63) | 4% |
| Non-CKD | 34 | 265/  28,278 | 1.39  (0.29, 7.17) | 0.66  (0.08, 3.95) | 0.92  (0.14, 5.25) | 0.48  (0.1, 1.65) | 0.68  (0.18, 1.95) | 1.36  (0.33, 6.79) | 14% |
| ACEi/ARB treatment | 21 | 1738/ 129,616 | **0.75**  **(0.63, 0.9)** | **0.69**  **(0.51, 0.92)** | 0.76  (0.58, 1.01) | 0.92  (0.72, 1.15) | 1.01  (0.82, 1.26) | 1.11  (0.82, 1.54) | 4% |
| Non-ACEi/ARB treatment | 41 | 264/ 37,965 | 0.48  (0.15, 1.49) | 0.34  (0.08, 1.21) | 0.42  (0.09, 1.41) | 0.72  (0.26, 1.54) | 0.91  (0.3, 1.57) | 1.24  (0.4, 3.1) | 18% |
| Cardiovascular outcome | 12 | 313/ 17,560 | 0.53  (0.06, 2.09) | 0.56  (0.02, 4.58) | 0.5  (0, 16.71) | 1.04  (0.12, 8.11) | 1.04  (0.02, 27.61) | 1.01  (0.01, 46.07) | 20% |
| Non-cardiovascular outcome | 49 | 1689/  150,024 | **0.73**  **(0.59, 0.88)** | **0.57**  **(0.4, 0.8)** | **0.74**  **(0.56, 0.95)** | 0.79  (0.61, 1.02) | 1.02  (0.86, 1.2) | 1.28  (0.93, 1.75) | 11% |

Odds ratio (95% credible interval) for comparisons are in cells between treatment. Bold and underlined cells are significant. OR< 1 favors treatment.

Abbreviation: Control represents either placebo or no treatment; DPP-4, dipeptidyl peptidase 4; GLP-1, glucagon-like peptide 1; and SGLT-2, sodium-glucose cotransporter 2.

**Appendix 9**

**Sensitivity analyses**

**Supplemental Table 10A.** Network meta-analysis for composite renal events after excluding trials with follow-up≤24 weeks or open-label trials

| **SGLT-2 inhibitor** | 1.09  (0.8, 1.42) | 0.99  (0.63, 1.52) | 0.87  (0.54, 1.34) |
| --- | --- | --- | --- |
| 0.88  (0.65, 1.27) | **Control** | 0.92  (0.64, 1.3) | 0.8  (0.56, 1.13) |
| 0.98  (0.61, 1.75) | 1.11  (0.76, 1.7) | **DPP-4 inhibitor** | 0.87  (0.54, 1.41) |
| 1.1  (0.7, 1.89) | 1.25  (0.88, 1.8) | 1.13  (0.66, 1.88) | **GLP-1 agonist** |

S1. Excluding trials with follow-up ≤24 weeks trials (51 trials, 9071 events, 156,282 patients, *I* ^2^ = 11%)

S2. Excluding trials with open-label trials (68 trials, 9162 events, 162,229 patients, *I* ^2^ = 10%)

**Supplemental Table 10B.** Network meta-analysis for AKI events after excluding trials with follow-up≤24 weeks or open-label trials

| **SGLT-2 inhibitor** | **1.35**  **(1.14, 1.61)** | **1.5**  **(1.16, 1.99)** | **1.32**  **(1.04, 1.69)** |
| --- | --- | --- | --- |
| **0.73**  **(0.6, 0.86)** | **Control** | 1.12  (0.91, 1.38) | 0.98  (0.82, 1.17) |
| **0.66**  **(0.49, 0.85)** | 0.9  (0.72, 1.11) | **DPP-4 inhibitor** | 0.88  (0.67, 1.14) |
| **0.74**  **(0.57, 0.92)** | 1.01  (0.85, 1.2) | 1.12  (0.86, 1.47) | **GLP-1 agonist** |

S1. Excluding trials with follow-up≤24 weeks trials (52 trials, 1978 events, 164,697 patients, *I* ^2^ = 16%)

S2. Excluding trials with open-label trials (56 trials, 1995 events, 164,088 patients, *I* ^2^ = 15%)

Results of network meta-analysis after excluding trials with follow-up ≤24 weeks and open-label trials were listed in the lower (S1) and upper triangle (S2). Comparisons should be read from left to right. Odds ratio (95% credible interval) for comparisons are in cells in common between column-defining and row-defining treatment. Bold and underlined cells are significan. OR <1 favors column-defining treatment.

Abbreviation: Control represents either placebo or no treatment; DPP-4, dipeptidyl peptidase 4; GLP-1, glucagon-like peptide 1; and SGLT-2, sodium-glucose cotransporter 2.

**Appendix 10**

**Meta-regression for network meta-analyses**

**Supplemental Table 11A.** Results of network meta-regression on composite renal events

| Drug | Covariate | OR drug vs Control | B (beta) of comparison | | |
| --- | --- | --- | --- | --- | --- |
|  |  | Odds ratio  (95% credible interval) | 2.5% | median | 97.5% |
| SGLT-2 inhibitor | BMI | 0.88 (0.66, 1.24) | -0.12 | 0.54 | 1.23 |
|  | Age | 1.00 (0.74, 1.39) | -1.07 | -0.36 | 0.29 |
|  | HbA1c | 0.93 (0.70, 1.27) | -1.08 | -0.17 | 0.79 |
|  | Duration of diabetes | 1.02 (0.75, 1.41) | -1.10 | -0.47 | 0.34 |
|  | eGFR | 0.58 (0.48, 0.78) | -0.65 | -0.09 | 0.59 |
| DPP-4 inhibitor | BMI | 0.92 (0.62, 1.34) | -1.37 | -0.11 | 1.34 |
|  | Age | 0.77 (0.47, 1.24) | -0.54 | 0.54 | 1.64 |
|  | HbA1c | 1.02 (0.67, 1.56) | -0.34 | 0.34 | 1.10 |
|  | Duration of diabetes | 0.79 (0.52, 1.16) | -0.47 | 0.61 | 1.76 |
|  | eGFR | 0.88 (0.67, 1.10) | **-1.41** | **-0.71** | **-0.08** |
| GLP-1 agonist | BMI | 0.82 (0.50, 1.37) | -1.71 | -0.19 | 1.25 |
|  | Age | 0.67 (0.39, 1.12) | -0.73 | 0.51 | 1.87 |
|  | HbA1c | 0.80 (0.55, 1.15) | -0.71 | -0.09 | 0.47 |
|  | Duration of diabetes | 0.76 (0.46, 1.23) | -1.18 | 0.15 | 1.48 |
|  | eGFR | 0.83 (0.67, 1.03) | -1.06 | -0.09 | 0.89 |

**Supplemental Table 11B.** Results of network meta-regression on acute kidney injury events

| Drug | Covariate | OR drug vs Control | B (beta) of comparison | | |
| --- | --- | --- | --- | --- | --- |
|  |  | Odds ratio  (95% credible interval) | 2.5% | median | 97.5% |
| SGLT-2 inhibitor | BMI | 0.73 (0.57, 0.89) | -1.47 | 0.22 | 2.36 |
|  | Age | 0.66 (0.47, 0.92) | -0.62 | 0.49 | 1.59 |
|  | HbA1c | 0.74 (0.62, 0.89) | -1.02 | -0.15 | 0.72 |
|  | Duration of diabetes | 0.75 (0.53, 1.16) | -1.12 | -0.11 | 0.80 |
|  | eGFR | 0.72 (0.58, 0.85) | -1.00 | -0.35 | 0.24 |
| DPP-4 inhibitor | BMI | 1.11 (0.90, 1.42) | -1.77 | -0.04 | 1.74 |
|  | Age | 1.15 (0.83, 1.56) | -0.84 | -0.08 | 0.77 |
|  | HbA1c | 1.15 (0.81, 1.60) | -0.71 | 0.04 | 0.76 |
|  | Duration of diabetes | 1.18 (0.93, 1.58) | -0.90 | -0.19 | 0.66 |
|  | eGFR | 1.23 (0.95, 1.62) | -0.29 | 0.34 | 0.99 |
| GLP-1 agonist | BMI | 0.91 (0.69, 1.16) | -0.53 | 0.59 | 2.10 |
|  | Age | 0.96 (0.82, 1.15) | -0.53 | -0.14 | 0.25 |
|  | HbA1c | 1.03 (0.85, 1.24) | -0.43 | -0.15 | 0.14 |
|  | Duration of diabetes | 1.01 (0.82, 1.28) | -0.71 | -0.22 | 0.29 |
|  | eGFR | 0.91 (0.74, 1.15) | -0.80 | 0.76 | 2.51 |

**Abbreviation**: BMI, body mass index, calculated as weight in kilograms

divided by height in meters squared; HbA1c, hemoglobin A1c; eGFR, estimated glomerular filtration rate; Control represents either placebo or no treatment; DPP-4, dipeptidyl peptidase 4; GLP-1, glucagon-like peptide 1; and SGLT-2, sodium-glucose cotransporter 2.

**Appendix 11**

**GRADE for the primary outcomes**

Table of reasons for downgrading

Based on the information presented above, we graded each network estimate using the criteria listed below.

1. Study limitations: When the contribution from low RoB comparisons was less than 30% and the contribution from moderate RoB comparisons was 70% or greater, we downgraded by one level.
2. Imprecision: We regarded 0.80 or 1.25 to be clinically significant thresholds for OR and downgraded the estimate if the OR point estimate is 1 or more and the lower limit of its CrI is less than 0.80; or if the OR point estimate is less than 1 and the upper limit of its CrI is greater than 1.25.

(3) Inconsistency: In this domain, we rated two concepts: heterogeneity and incoherence (inconsistency). We used *I* ^2^ statistic to assess heterogeneity in the study. We downgraded by one level when the *I* ^2^ statistic values over 50%. We looked at the results of node splitting for inconsistency and downgraded the comparisons with significant inconsistency (p< 0.10). The design-by-treatment test is used to assess the inconsistency of the ranking results.

1. Indirectness: We have assured transitivity in our network by comparing the distribution of clinical variables that could act as effect modifiers across treatment comparisons. The subgroup analyses, sensitivity analyses and network metaregressions were used to evaluate indirectness.
2. Publication bias: The comparison-adjusted funnel plot was used to evaluate overall publication bias, and the funnel plot by Egger test was used to assess direct comparison bias. But for AKI events, the majority data comes from adverse event reporting rather than trial data, so we decided to downgrade all the included studies of this outcome for potential publication bias by one level.

**Supplemental Table 12A.** Evaluation of the quality of evidence using GRADE framework for composite renal events

| **Comparison** | **Study limitation** | **Imprecision** | **Heterogeneity and inconsistency** | **Indirectness** | **Publication bias** | **GRADE** |
| --- | --- | --- | --- | --- | --- | --- |
| SGLT-2i vs Control | No downgrade | No downgrade  (OR= 0.92, 95%CI: 0.7 to 1.24) | Downgrade because heterogeneity.  Severe heterogeneity according to *I* ^2^ (70.1%) in direct comparisons.  No inconsistency between direct and indirect estimate (Node-split p=0.817). | The treatment effects were significantly influenced by clinical modifiers in the subgroup analyses. | The funnel plot for the direct comparison suggest that there might be small-study effects (Egger test, P= 0.004). | Very low (Downgrade by three levels due to heterogeneity, indirectness and publication bias). |
| SGLT-2i vs DPP-4i | No downgrade | Downgrade  because point  estimate =1.0  but lower limit  <0.80  (OR= 1.0, 95%CI: 0.66 to 1.59) | No downgrade  Mild heterogeneity according to *I* ^2^ (0.0%) in direct comparisons.  No inconsistency between direct and indirect estimate (Node-split p=0.867) | The treatment effects were significantly influenced by clinical modifiers in the subgroup analyses. | Undetectable by routine methods. | Low (Downgrade by two levels due to imprecision and indirectness). |
| SGLT-2i vs GLP-1a | No downgrade | Downgrade  because point  estimate >1.0  but lower limit  <0.80  (OR= 1.09, 95%CI: 0.70 to 1.56) | No downgrade  Mild heterogeneity according to *I* ^2^ (0.0%) in direct comparisons.  No inconsistency between direct and indirect estimate (Node-split p=0.743) | The treatment effects were significantly influenced by clinical modifiers in the subgroup analyses. | The funnel plot for the direct comparison is not suggestive of any dominant publication bias. | Low (Downgrade by two levels due to imprecision and indirectness) |
| Control vs DPP-4i | No downgrade | No downgrade  (OR= 1.26, 95%CI: 0.9 to 1.8) | No downgrade  Mild heterogeneity according to *I* ^2^ (0.0%) in direct comparisons.  No inconsistency between direct and indirect estimate (Node-split p=0.472) | The treatment effects were significantly influenced by clinical modifiers (such as eGFR). | The funnel plot for the direct comparison is not suggestive of any dominant publication bias. | Moderate (Downgrade by one level due to indirectness). |
| Control vs GLP-1a | No downgrade | Downgrade  because point  estimate >1.0  but lower limit  <0.80  (OR= 1.15, 95%CI: 0.72 to 1.89) | No downgrade  Mild heterogeneity according to *I* ^2^ (0.0%) in direct comparisons.  No inconsistency between direct and indirect estimate (Node-split p=0.786) | The treatment effects were significantly influenced by clinical modifiers in the subgroup analyses. | Undetectable by routine method. | Low (Downgrade by two levels due to imprecision and indirectness). |
| DPP-4i vs GLP-1a | No downgrade | Downgrade  because point  estimate >1.0  but lower limit  <0.80  (OR= 1.16, 95%CI: 0.76 to 1.88) | No downgrade  Mild heterogeneity according to *I* ^2^ (0.0%) in direct comparisons.  Only indirect comparison and no node-splitting inconsistency. | No serious  Indirectness. | Undetectable by routine method. | Moderate (Downgrade by one level due to indirectness). |
| Ranking of treatment | No downgrade | No downgrade  SUCRA plots suggested the precision in a ranking of treatments. | No downgrade  Mild heterogeneity in network meta-analyses according to global *I* ^2^ (10.0%).  No significant inconsistency in test of global inconsistency (P = 0.902). | The treatment effects were significantly influenced by clinical modifiers in the subgroup analyses. | Comparison-adjusted funnel plot for the network is not suggestive of any dominant publication bias. | Moderate (Downgrade by one level due to indirectness). |

**Supplemental Table 12B.** Evaluation of the quality of evidence using GRADE framework for AKI events.

| **Comparison** | **Study limitation** | **Imprecision** | **Heterogeneity and inconsistency** | **Indirectness** | **Publication bias** | **GRADE** |
| --- | --- | --- | --- | --- | --- | --- |
| SGLT-2i vs Control | No downgrade | No downgrade  (OR= 0.74, 95%CI: 0.62 to 0.87) | Mild heterogeneity according to *I* ^2^ (0.0%) in direct comparisons.  There is inconsistency between direct and indirect estimate (Node-split p=0.000). | No serious  Indirectness. | Downgrade | Low (Downgrade by two levels due to inconsistency and publication bias). |
| SGLT-2i vs DPP-4i | No downgrade | No downgrade (OR= 0.67, 95%CI: 0.50 to 0.86) | Mild heterogeneity according to *I* ^2^ (0.0%) in direct comparisons.  There is inconsistency between direct and indirect estimate (Node-split p=0.013) | No serious  Indirectness. | Downgrade | Low (Downgrade by two levels due to inconsistency and publication bias). |
| Control vs DPP-4i | No downgrade | No downgrade (OR= 0.90, 95%CI: 0.72 to 1.1) | Mild heterogeneity according to *I* ^2^ (0.0%) in direct comparisons.  No inconsistency between direct and indirect estimate (Node-split p=0.612) | No serious  Indirectness. | Downgrade | Moderate (Downgrade by one level due to publication bias). |
| Control vs GLP-1a | No downgrade | No downgrade (OR=1.0, 95%CI: 0.86 to 1.2) | Mild heterogeneity according to *I* ^2^ (0.0%) in direct comparisons.  No inconsistency between direct and indirect estimate (Node-split p=0.733) | No serious  Indirectness. | Downgrade | Moderate (Downgrade by one level due to publication bias). |
| DPP-4i vs GLP-1a | No downgrade | No downgrade (OR= 1.10, 95%CI: 0.88 to 1.50) | Mild heterogeneity according to *I* ^2^ (0.0%) in direct comparisons.  No inconsistency between direct and indirect estimate (Node-split p=0.335) | No serious  Indirectness. | Downgrade | Moderate (Downgrade by one level due to publication bias). |
| SGLT-2i vs GLP-1a | No downgrade | No downgrade (OR= 0.76, 95%CI: 0.59 to 0.96) | Mild heterogeneity according to *I* ^2^ (0.0%) in direct comparisons.  No inconsistency between direct and indirect estimate (Node-split p=0.681) | No serious  Indirectness. | Downgrade | Moderate (Downgrade by one level due to publication bias). |
| Ranking of treatment | No downgrade | SUCRA plots suggested the precision in a ranking of treatments. | Mild heterogeneity in network meta-analyses according to global *I* ^2^ (15.0%).  No significant inconsistency in test of global inconsistency (P = 0.902). | No serious  Indirectness. | Downgrade | Moderate (Downgrade by one level due to publication bias). |

**Appendix 12**

**Drug doses and the code of network meta-analysis for main results in R software**

**Supplemental 2**. Drug doses

Data only extracted and analyzed for study groups receiving either marketed drug

doses or doses evaluated in phase 3 clinical trials

**SGLT-2 inhibitors**

Empagliflozin total daily dose 10 to 25mg PO

Canagliflozin total daily dose 100 to 300mg PO

Dapagliflozin total daily dose 5 to 10mg PO

Ipragliflozin total daily dose 50 to 100mg PO

Luseogliflozin total daily dose 2.5 to 5mg PO

Ertugliflozin total daily dose 5 to 15mg PO

Bexagliflozin total daily dose 20mg

Remogliflozin excluded as no phase 3/marketing dose

Tofogliflozin excluded as no phase 3/marketing dose

**GLP-1 agonists**

Dulaglutide 0.75-1.5mg SC once weekly

Semaglutide 0.5-1mg SC once weekly

Oral Semaglutide total daily dose 3 to 14mg PO

Liraglutide 0.6 to 1.8mg SC daily

Lixisenatide 5 to 20mcg SC daily

Exenatide 5 to 10mcg SC twice daily

Albiglutide 30 to 50mg SC once weekly

**DPP-4 inhibitors**

Alogliptin total daily dose 6.25 to 25mg PO

Saxagliptin total daily dose 2.5 to 5mg PO

Sitagliptin total daily dose 25 to 100mg PO

Linagliptin total daily dose 5mg PO

Vildagliptin total daily dose 50 to 100mg PO

Omarigliptin 12.5 to 25mg once a week PO

Tenelegliptin excluded as no phase 3/marketing dose

Gemigliptin excluded as no phase 3/marketing dose

Evogliptin excluded as no phase 3/marketing dose

**Supplemental 3.1** The code of network meta-analysis for composite renal events in R software

Code :

model {

# Likelihood for arm-based data

for (i in studies.a) {

for (k in 1:na[i]) {

logit(p[i, k]) <- mu[i] + delta[i, k]

r[i, k] ~ dbin(p[i, k], n[i, k])

rhat[i, k] <- p[i, k] * n[i, k]

dev[i, k] <- 2 *

(r[i, k] * (log(r[i, k]) - log(rhat[i, k])) +

(n[i, k]-r[i, k]) * (log(n[i, k] - r[i, k]) - log(n[i, k] - rhat[i, k])))

}

}

# Likelihood for contrast-based data (univariate for 2-arm trials)

## OMITTED

# Likelihood for contrast-based data (multivariate for multi-arm trials)

## OMITTED

# Random effects model

for (i in studies) {

# Study-level relative effects

w[i, 1] <- 0

delta[i, 1] <- 0

for (k in 2:na[i]) { # parameterize multi-arm trials using a trick to avoid dmnorm

delta[i, k] ~ dnorm(md[i, k], taud[i, k])

md[i, k] <- d[t[i, 1], t[i, k]] + sw[i, k]

taud[i, k] <- tau.d * 2 * (k - 1) / k

w[i, k] <- delta[i, k] - (d[t[i, 1], t[i, k]])

sw[i, k] <- sum(w[i, 1:(k-1)]) / (k - 1)

}

}

# Random effects variance prior

sd.d ~ dunif(0, om.scale)

tau.d <- pow(sd.d, -2)

# Relative effect matrix

d[1, 1] <- 0

d[1, 2] <- -d.2.1

d[1, 3] <- -d.2.1 + d.2.3

d[1, 4] <- -d.2.1 + d.2.4

for (i in 2:nt) {

for (j in 1:nt) {

d[i, j] <- d[1, j] - d[1, i]

}

}

prior.prec <- pow(re.prior.sd, -2)

# Study baseline priors

for (i in studies.a) {

mu[i] ~ dnorm(0, prior.prec)

}

# Effect parameter priors

d.2.1 ~ dnorm(0, prior.prec)

d.2.3 ~ dnorm(0, prior.prec)

d.2.4 ~ dnorm(0, prior.prec)

}

**Supplemental 3.2** The code of network meta-analysis for AKI events in R software

Code :

model {

# Likelihood for arm-based data

for (i in studies.a) {

for (k in 1:na[i]) {

logit(p[i, k]) <- mu[i] + delta[i, k]

r[i, k] ~ dbin(p[i, k], n[i, k])

rhat[i, k] <- p[i, k] * n[i, k]

dev[i, k] <- 2 *

(r[i, k] * (log(r[i, k]) - log(rhat[i, k])) +

(n[i, k]-r[i, k]) * (log(n[i, k] - r[i, k]) - log(n[i, k] - rhat[i, k])))

}

}

# Likelihood for contrast-based data (univariate for 2-arm trials)

## OMITTED

# Likelihood for contrast-based data (multivariate for multi-arm trials)

## OMITTED

# Random effects model

for (i in studies) {

# Study-level relative effects

w[i, 1] <- 0

delta[i, 1] <- 0

for (k in 2:na[i]) { # parameterize multi-arm trials using a trick to avoid dmnorm

delta[i, k] ~ dnorm(md[i, k], taud[i, k])

md[i, k] <- d[t[i, 1], t[i, k]] + sw[i, k]

taud[i, k] <- tau.d * 2 * (k - 1) / k

w[i, k] <- delta[i, k] - (d[t[i, 1], t[i, k]])

sw[i, k] <- sum(w[i, 1:(k-1)]) / (k - 1)

}

}

# Random effects variance prior

sd.d ~ dunif(0, om.scale)

tau.d <- pow(sd.d, -2)

# Relative effect matrix

d[1, 1] <- 0

d[1, 2] <- d.1.2

d[1, 3] <- d.1.3

d[1, 4] <- d.1.4

for (i in 2:nt) {

for (j in 1:nt) {

d[i, j] <- d[1, j] - d[1, i]

}

}

prior.prec <- pow(re.prior.sd, -2)

# Study baseline priors

for (i in studies.a) {

mu[i] ~ dnorm(0, prior.prec)

}

# Effect parameter priors

d.1.2 ~ dnorm(0, prior.prec)

d.1.3 ~ dnorm(0, prior.prec)

d.1.4 ~ dnorm(0, prior.prec)

}

**Appendix 13**

**Extract form**

# **Data extraction form**

Reviewers name

**Study ID – First Author, Date of Publication, Title, Place of Publication**

#### Study design

Parallel group Crossover Other (describe)

Is the study randomised?

Is the study double blind? ________________________

##### **Participants**

Diagnosis

|  | Intervention1 | Intervention2 | Control |
| --- | --- | --- | --- |
| Diagnostic criteria of patients included in the trial |  | | |
| Age (mean, sd) |  |  |  |
| Sex (male n (%)) |  |  |  |
| Race (Primary)% |  |  |  |
| eGFR (mean, sd (ml/min/1.73 m2 )) |  |  |  |
| Pre-existing CKD (%) |  |  |  |
| Pre-existing CVD (%) |  |  |  |
| HbA 1c (mean, sd (%) ) |  |  |  |
| Mean BMI (mean, sd (kg/m2)) |  |  |  |
| Background therapy /ACEi /ARB (%) |  |  |  |
| Number of participants entered in trial |  |  |  |
| Follow-up (weeks) |  |  |  |
| Duration of diabetes (mean, sd (years)) |  |  |  |
| Background treatments |  | | |

###### Intervention

Does intervention fulfil criteria for inclusion?

|  | Intervention1 | Intervention2 | Control |
| --- | --- | --- | --- |
| Type of intervention |  |  |  |
| Formulation |  |  |  |
| Timing/frequency |  |  |  |
| Duration |  |  |  |
| Setting |  |  |  |
| Length of follow-up |  |  |  |

# Were treatment groups comparable at baseline?

Yes No Don’t know

**Include study?**

No (give reasons for exclusion)

The Cochrane Collaboration’s tool for assessing risk of bias

| **Domain** | **Description** | **Review authors’ judgement** |
| --- | --- | --- |
| **Sequence generation.** |  | Was the allocation sequence adequately generated?  YES/NO/UNCLEAR |
| **Allocation concealment.** |  | Was allocation adequately concealed?  YES/NO/UNCLEAR |
| **Blinding of participants, personnel and outcome assessors** *Assessments should be made for each main outcome (or class of outcomes).* |  | Was knowledge of the allocated intervention adequately prevented during the study?  YES/NO/UNCLEAR |
| **Incomplete outcome data** *Assessments should be made for each main outcome (or class of outcomes).* |  | Were incomplete outcome data adequately addressed?  YES/NO/UNCLEAR |
| **Selective outcome reporting.** |  | Are reports of the study free of suggestion of selective outcome reporting?  YES/NO/UNCLEAR |
| **Other sources of bias.** |  | Was the study apparently free of other problems that could put it at a high risk of bias?  YES/NO/UNCLEAR |

Summary assessment for bias within study:

LOW/UNCLEAR/HIGH

## Outcomes

# Primary outcome

Outcome

Time of measurement

|  | Number of events | Total number | Notes/Additional data/Missing data |
| --- | --- | --- | --- |
| Intervention 1 |  |  |  |
| Intervention 2 |  |  |  |
| Control |  |  |  |

###### Secondary Outcomes

Outcome

Time of measurement

|  | Number of events | Total number | Notes/Additional data/Missing data |
| --- | --- | --- | --- |
| Intervention 1 |  |  |  |
| Intervention 2 |  |  |  |
| Control |  |  |  |

**5. Details of any additional outcomes used in published papers but not specified in the protocol**

**6. Text entry for main conclusions of the trial**

**Appendix 14**

**References for included trials**

**Supplemental references**

1. Stenlöf K, Cefalu WT, Kim KA, *et al*. Efficacy and safety of canagliflozin monotherapy in subjects with type 2 diabetes mellitus inadequately controlled with diet and exercise. *Diabetes, obesity & metabolism*. 2013;15(4):372-382.
2. Yale JF, Bakris G, Cariou B, et al. Efficacy and safety of canagliflozin over 52 weeks in patients with type 2 diabetes mellitus and chronic kidney disease. *Diabetes, obesity & metabolism*. 2014;16(10):1016-1027.
3. Bode B, Stenlof K, Harris S, *et al*. [Long-term efficacy and safety of canagliflozin over 104 weeks in patients aged 55-80 years with type 2 diabetes.](https://pubmed02.jp.goftp.xyz/25495720/) *Diabetes, obesity & metabolism*. 2015;17(3):294-303.
4. Mahaffey KW, Jardine MJ, Bompoint S, *et al.* Canagliflozin and Cardiovascular and Renal Outcomes in Type 2 Diabetes and Chronic Kidney Disease in Primary and Secondary Cardiovascular Prevention Groups: results from the Randomized CREDENCE Trial. *Circulation*. 2019;140(9):739-750.
5. Perkovic V, de Zeeuw D, Mahaffey KW, *et al*. Canagliflozin and renal outcomes in type 2 diabetes: results from the CANVAS Program randomised clinical trials. *Lancet Diabetes Endocrinol*. 2018;6(9):691-704.
6. Rosenstock J, Chuck L, González-Ortiz M, *et al*. Initial Combination Therapy With Canagliflozin Plus Metformin Versus Each Component as Monotherapy for Drug-Naïve Type 2 Diabetes. *Diabetes care*. 2016;39(3):353-362.
7. Qiu R, Capuano G, Meininger G. Efficacy and safety of twice-daily treatment with canagliflozin, a sodium glucose co-transporter 2 inhibitor, added on to metformin monotherapy in patients with type 2 diabetes mellitus. *Journal of clinical and translational endocrinology*. 2014;1(2):54-60.
8. Allegretti AS, Zhang W, Zhou W*, et al*. Safety and Effectiveness of Bexagliflozin in Patients With Type 2 Diabetes Mellitus and Stage 3a/3b CKD. *American Journal of Kidney Diseases*. 2019;74(3):328-337.
9. Grunberger G, Camp S, Johnson J, *et al*. Ertugliflozin in Patients with Stage 3 Chronic Kidney Disease and Type 2 Diabetes Mellitus: the VERTIS RENAL Randomized Study. *Diabetes therapy*. 2018;9(1):49-66.
10. Haneda M, Seino Y, Inagaki N, *et al*. Influence of Renal Function on the 52-Week Efficacy and Safety of the Sodium Glucose Cotransporter 2 Inhibitor Luseogliflozin in Japanese Patients with Type 2 Diabetes Mellitus. *Clinical therapeutics*. 2016;38(1):66-88.e20.
11. Monteiro P, Bergenstal RM, Toural E, *et al*. Efficacy and safety of empagliflozin in older patients in the EMPA-REG OUTCOME® trial*. Age and ageing*. 2019;48(6):859-866.
12. Barnett AH, Mithal A, Manassie J, *et al*. Efficacy and safety of empagliflozin added to existing antidiabetes treatment in patients with type 2 diabetes and chronic kidney disease: a randomised, double-blind, placebo-controlled trial.*The lancet. Diabetes & endocrinology*. 2014;2(5):369-384.
13. Rosenstock J, Jelaska A, Zeller C, *et al*. Impact of empagliflozin added on to basal insulin in type 2 diabetes inadequately controlled on basal insulin: a 78-week randomized, double-blind, placebo-controlled trial. *Diabetes, obesity & metabolism*. 2015;17(10):936-948.
14. Rosenstock J, Jelaska A, Frappin G, *et al*. Improved glucose control with weight loss, lower insulin doses, and no increased hypoglycemia with empagliflozin added to titrated multiple daily injections of insulin in obese inadequately controlled type 2 diabetes. *Diabetes care*. 2014;37(7):1815-1823.
15. Häring HU, Merker L, Seewaldt-Becker E, *et al*. Empagliflozin as add-on to metformin in patients with type 2 diabetes: a 24-week, randomized, double-blind, placebo-controlled trial. *Diabetes care*. 2014;37(6):1650-1659.
16. Haering HU, Merker L, Christiansen AV, *et al*. Empagliflozin as add-on to metformin plus sulphonylurea in patients with type 2 diabetes. *Diabetes research and clinical practice*. 2015;110(1):82-90.
17. Roden M, Merker L, Christiansen AV, *et al.* Safety, tolerability and effects on cardiometabolic risk factors of empagliflozin monotherapy in drug-naive patients with type 2 diabetes: a double-blind extension of a Phase III randomized controlled trial. *Cardiovascular diabetology*. 2015;14:154.
18. Søfteland E, Meier JJ, Vangen B, *et al*. Empagliflozin as Add-on Therapy in Patients With Type 2 Diabetes Inadequately Controlled With Linagliptin and Metformin: a 24-Week Randomized, Double-Blind, Parallel-Group Trial. *Diabetes care*. 2017;40(2):201-209.
19. Kohan DE, Fioretto P, Tang W, List JF. Long-term study of patients with type 2

diabetes and moderate renal impairment shows that dapagliflozin reduces weight

and blood pressure but does not improve glycemic control. *Kidney International*. 2014;85(4):962-971.

1. Cefalu WT, Leiter LA, De Bruin TW, *et al*. Dapagliflozin's effects on glycemia and cardiovascular risk factors in high-risk patients with type 2 diabetes: A 24-week, multicenter, randomized, double-blind, placebo-controlled study with a 28-week extension. *Diabetes Care*. 2015;38(7):1218-1227.
2. Leiter LA, Cefalu WT, De Bruin TW, *et al*. Dapagliflozin added to usual care in individuals with type 2 diabetes mellitus with preexisting cardiovascular disease: A 24-week, multicenter, randomized, double-blind, placebo-controlled study with a 28-week extension. *Journal of the American Geriatrics Society*. 2014;62(7):1252-1262.
3. Kaku K, Kiyosue A, Inoue S, *et al*. Japanese patients with type 2 diabetes inadequately controlled by diet and exercise. *Diabetes.obesity & metabolism*. 2014;16(11):1102-1110.
4. Mosenzon O, Wiviott SD, Cahn A, *et al*. Effects of dapagliflozin on development and progression of kidney disease in patients with type 2 diabetes: an analysis from the DECLARE–TIMI 58 randomised trial. *The lancet diabetes and endocrinology*. 2019 Aug;7(8):606-617.
5. Pollock C, Stefánsson B, Reyner D, *et al*. Albuminuria-lowering effect of dapagliflozin alone and in combination with saxagliptin and effect of dapagliflozin and saxagliptin on glycaemic control in patients with type 2 diabetes and chronic kidney disease (DELIGHT): a randomised, double-blind, placebo-controlled trial. *The Lancet Diabetes and Endocrinology*. 2019;7(6):429-441.
6. Heerspink HJ, Johnsson E, Gause-Nilsson I, *et al*. Dapagliflozin reduces albuminuria in patients with diabetes and hypertension receiving renin-angiotensin blockers. *Diabetes, obesity & metabolism*. 2016;18(6):590-597.
7. Bailey CJ, Gross JL, Hennicken D, *et a*l. Dapagliflozin add-on to metformin in type 2 diabetes inadequately controlled with metformin: a randomized, double-blind, placebo-controlled 102-week trial. *BMC medicine*. 2013;11:43.
8. Bailey CJ, Iqbal N, T'Joen C, List JF. Dapagliflozin monotherapy in drug-naive patients with diabetes: a randomized-controlled trial of low-dose range. *Diabetes, obesity & metabolism.* 2012;14(10):951-959.
9. Bailey CJ, Morales Villegas EC, Woo V, *et al*. Efficacy and safety of dapagliflozin monotherapy in people with Type 2 diabetes: a randomized double-blind placebo-controlled 102-week trial. *Diabetic medicine*. 2015;32(4):531-541.
10. Araki E, Onishi Y, Asano M, *et al*. Efficacy and safety of dapagliflozin in addition to insulin therapy in Japanese patients with type 2 diabetes: results of the interim analysis of 16-week double-blind treatment period. *Journal of diabetes investigation*. 2016;7(4):555-564.
11. Wilding JP, Norwood P, T'Joen C*, et al*. A study of dapagliflozin in patients with type 2 diabetes receiving high doses of insulin plus insulin sensitizers: Applicability of a novel insulin-independent treatment. *Diabetes Care*.2009;32(9):1656-1662.
12. Yang W, Han P, Min KW, *et al*. Efficacy and safety of dapagliflozin in Asian patients with type 2 diabetes after metformin failure: a randomized controlled trial. *Journal of diabetes*. 2016;8(6):796-808.
13. Ji L, Ma J, Li H, *et al*. Dapagliflozin as monotherapy in drug-naive Asian patients with type 2 diabetes mellitus: a randomized, blinded, prospective phase III study. *Clinical therapeutics*. 2014;36(1):84-100.e9.
14. Schumm-Draeger PM, Burgess L, Korányi L, *et al*. Twice-daily dapagliflozin co-administered with metformin in type 2 diabetes: a 16-week randomized, placebo-controlled clinical trial. *Diabetes, obesity & metabolism*. 2015;17(1):42-51.
15. Matthaei S, Bowering K, Rohwedder K*, et al*. Durability and tolerability of dapagliflozin over 52weeks as add-on to metformin and sulphonylurea in type 2 diabetes. *Diabetes, obesity & metabolism*. 2015;17(11):1075-1084.
16. Mathieu C, Ranetti AE, Li D, *et al*. Randomized, Double-Blind, Phase 3 Trial of Triple Therapy With Dapagliflozin Add-on to Saxagliptin Plus Metformin in Type 2 Diabetes*. Diabetes care*. 2015;38(11):2009-2017.
17. Bolinder J, Ljunggren Ö, Johansson L ,*et al*. Dapagliflozin maintains glycaemic control while reducing weight and body fat mass over 2 years in patients with type 2 diabetes mellitus inadequately controlled on metformin. *Diabetes, obesity & metabolism*. 2014;16(2):159-169.
18. Jabbour SA, Hardy E, Sugg J, Parikh S. Dapagliflozin is effective as add-on therapy to sitagliptin with or without metformin: a 24-week, multicenter, randomized, double-blind, placebo-controlled study. *Diabetes care*. 2014;37(3):740-750.
19. Wilding JP, Woo V, Rohwedder K, Sugg J, Parikh S. Dapagliflozin in patients with type 2 diabetes receiving high doses of insulin: efficacy and safety over 2 years. *Diabetes, obesity & metabolism*. 2014;16(2):124-136.
20. Strojek K, Yoon KH, Hruba V, *et al*. Dapagliflozin Added to Glimepiride in Patients with Type 2 Diabetes Mellitus Sustains Glycemic Control and Weight Loss Over 48 Weeks: a Randomized, Double-Blind, Parallel-Group, Placebo-Controlled Trial. *Diabetes therapy*. 2014;5(1):267-283.
21. White WB, Cannon CP, HellerSR, *et al*. Alogliptin after acute coronary syndrome in patients with type 2 diabetes. *New England journal of medicine*. 2013;369(14):1327-1335.
22. Lewin AJ, Arvay L, Liu D, Patel S, von Eynatten M, Woerle HJ. Efficacy and Tolerability of Linagliptin Added to a Sulfonylurea Regimen in Patients With Inadequately Controlled Type 2 Diabetes Mellitus: An 18-Week, Multicenter, Randomized, Double-Blind, Placebo-Controlled Trial. Clinical therapeutics. 2012;34(9):1909-19.e15.
23. NCT02897349. [https://clinicaltrials.gov/ct2/show/NCT02897349.](https://clinicaltrials.gov/ct2/show/NCT00849017.)
24. Yki-Järvinen H, Rosenstock J, Durán-Garcia, *et al*. Effects of adding linagliptin to basal insulin regimen for inadequately controlled type 2 diabetes: a ≥52-week randomized, double-blind study. *Diabetes care*. 2013;36(12):3875-3881.
25. Ledesma G, Umpierrez GE, Morley JE, *et al*. Efficacy and safety of linagliptin to improve glucose control in older people with type 2 diabetes on stable insulin therapy: a randomized trial. *Diabetes, obesity & metabolism*. 2019;21(11):2465-2473.
26. Cooper ME, Perkovic V, Groop PH, *et al*. Hemodynamic effects of the dipeptidyl peptidase-4 inhibitor linagliptin with renin-angiotensin system inhibitors in type 2 diabetic patients with albuminuria. *Journal of hypertension*. 2019;37(6):1294-1300.
27. Inagaki N, Yang W, Watada H, *et al*. Linagliptin and cardiorenal outcomes in Asians with type 2 diabetes mellitus and established cardiovascular and/or kidney disease: subgroup analysis of the randomized CARMELINA® trial. *Diabetology International*. 2019;11(2):129-141.
28. McGill JB, Sloan L, Newman J, *et al*. Long-term efficacy and safety of linagliptin in patients with type 2 diabetes and severe renal impairment: a 1-year, randomized, double-blind, placebo-controlled study. *Diabetes care*. 2013;36(2):237-244.
29. Owens DR, Swallow R, Dugi KA, Woerle HJ. Efficacy and safety of linagliptin in persons with type 2 diabetes inadequately controlled by a combination of metformin and sulphonylurea: a 24-week randomized study. *Diabetic medicine.* 2011;28(11):1352-1361.
30. Matthews DR, Paldánius PM, Proot P, *et al*. Glycaemic durability of an early combination therapy with vildagliptin and metformin versus sequential metformin monotherapy in newly diagnosed type 2 diabetes (VERIFY): a 5-year, multicentre, randomised, double-blind trial. *The Lancet*. 2019;394(10208):1519-1529.
31. Frias JP, Zimmer Z, Lam RLH, *et al*. Double-blind, randomized clinical trial assessing the efficacy and safety of early initiation of sitagliptin during metformin uptitration in the treatment of patients with type 2 diabetes: the CompoSIT-M study. *Diabetes, obesity & metabolism*. 2019;21(5):1128-1135.
32. Barzilai N, Guo H, Mahoney EM, *et al*. Efficacy and tolerability of sitagliptin monotherapy in elderly patients with type 2 diabetes: a randomized, double-blind, placebo-controlled trial. *Current medical research and opinion*. 2011;27(5):1049-1058.
33. Shankar RR,Bao Y,Han P*, et al*. Sitagliptin added to stable insulin therapy with or without metformin in Chinese patients with type 2 diabetes. *Journal of diabetes investigation*. 2017;8(3):321-329.
34. Green JB, Bethel MA, Armstrong PW, *et al.* Effect of Sitagliptin on Cardiovascular Outcomes in Type 2 Diabetes. *N Engl J Med*. 2015;373(3):232-42.
35. Gantz I, Chen M, Suryawanshi S, *et al*. A randomized, placebo-controlled study of the cardiovascular safety of the once-weekly DPP-4 inhibitor omarigliptin in patients with type 2 diabetes mellitus. *Cardiovascular diabetology*. 2017;16(1):112.
36. Chacra A, Gantz I, Mendizabal G, *et al*. A randomised, double-blind, trial of the safety and efficacy of omarigliptin (a once-weekly DPP-4 inhibitor) in subjects with type 2 diabetes and renal impairment. *International journal of clinical practice*. 2017;71(6):e12955.
37. DeFronzo RA, Hissa MN, Garber AJ, *et al.* The efficacy and safety of saxagliptin when added to metformin therapy in patients with inadequately controlled type 2 diabetes with metformin alone. *Diabetes Care*. 2009;32(9):1649-1655.
38. Barnett AH, Charbonnel B, Li J, *et al*. Saxagliptin add-on therapy to insulin with or without metformin for type 2 diabetes mellitus: 52-week safety and efficacy. *Clinical drug investigation*. 2013;33(10):707-717.
39. Müller-Wieland D, Kellerer M, Cypryk K*, et al*. Efficacy and safety of dapagliflozin or dapagliflozin plus saxagliptin versus glimepiride as add-on to metformin in patients with type 2 diabetes. *Diabetes, obesity & metabolism*. 2018;20(11):2598-2607.
40. Dou J, Ma J, Liu J, *et al*. Efficacy and safety of saxagliptin in combination with metformin as initial therapy in Chinese patients with type 2 diabetes: results from the START study, a multicentre, randomized, double-blind, active-controlled, phase 3 trial. *Diabetes, obesity & metabolism*. 2018;20(3):590-598.
41. [Nowicki M](https://pubmed.th.dataotetao.com/pubmed/?term=Nowicki M[Author]&cauthor=true&cauthor_uid=21332627), [Rychlik I](https://pubmed.th.dataotetao.com/pubmed/?term=Rychlik I[Author]&cauthor=true&cauthor_uid=21332627), [Haller H](https://pubmed.th.dataotetao.com/pubmed/?term=Haller H[Author]&cauthor=true&cauthor_uid=21332627), [Warren ML](https://pubmed.th.dataotetao.com/pubmed/?term=Warren ML[Author]&cauthor=true&cauthor_uid=21332627), [Suchower L](https://pubmed.th.dataotetao.com/pubmed/?term=Suchower L[Author]&cauthor=true&cauthor_uid=21332627), [Gause-Nilsson I](https://pubmed.th.dataotetao.com/pubmed/?term=Gause-Nilsson I[Author]&cauthor=true&cauthor_uid=21332627). Saxagliptin improves glycaemic control and is well tolerated in patients with type 2 diabetes mellitus and renal impairment. [*Diabetes Obes Metab*.](https://pubmed.th.dataotetao.com/pubmed/21332627) 2011;13(6):523-32.
42. [Mosenzon O](https://pubmed.th.dataotetao.com/pubmed/?term=Mosenzon O[Author]&cauthor=true&cauthor_uid=27797925), [Leibowitz G](https://pubmed.th.dataotetao.com/pubmed/?term=Leibowitz G[Author]&cauthor=true&cauthor_uid=27797925), [Bhatt DL](https://pubmed.th.dataotetao.com/pubmed/?term=Bhatt DL[Author]&cauthor=true&cauthor_uid=27797925), [Cahn A](https://pubmed.th.dataotetao.com/pubmed/?term=Cahn A[Author]&cauthor=true&cauthor_uid=27797925), [Hirshberg B](https://pubmed.th.dataotetao.com/pubmed/?term=Hirshberg B[Author]&cauthor=true&cauthor_uid=27797925), [Wei C](https://pubmed.th.dataotetao.com/pubmed/?term=Wei C[Author]&cauthor=true&cauthor_uid=27797925), [Im K](https://pubmed.th.dataotetao.com/pubmed/?term=Im K[Author]&cauthor=true&cauthor_uid=27797925), [Rozenberg A](https://pubmed.th.dataotetao.com/pubmed/?term=Rozenberg A[Author]&cauthor=true&cauthor_uid=27797925), [Yanuv I](https://pubmed.th.dataotetao.com/pubmed/?term=Yanuv I[Author]&cauthor=true&cauthor_uid=27797925), [Stahre C](https://pubmed.th.dataotetao.com/pubmed/?term=Stahre C[Author]&cauthor=true&cauthor_uid=27797925), [Ray KK](https://pubmed.th.dataotetao.com/pubmed/?term=Ray KK[Author]&cauthor=true&cauthor_uid=27797925), [Iqbal N](https://pubmed.th.dataotetao.com/pubmed/?term=Iqbal N[Author]&cauthor=true&cauthor_uid=27797925), [Braunwald E](https://pubmed.th.dataotetao.com/pubmed/?term=Braunwald E[Author]&cauthor=true&cauthor_uid=27797925), [Scirica BM](https://pubmed.th.dataotetao.com/pubmed/?term=Scirica BM[Author]&cauthor=true&cauthor_uid=27797925), [Raz I](https://pubmed.th.dataotetao.com/pubmed/?term=Raz I[Author]&cauthor=true&cauthor_uid=27797925). Effect of Saxagliptin on Renal Outcomes in the SAVOR-TIMI 53 Trial. [*Diabetes Care*.](https://pubmed.th.dataotetao.com/pubmed/27797925) 2017 Jan;40(1):69-76.
43. Aroda VR, Rosenstock J, Wysham C, *et al*. Efficacy and Safety of LixiLan, a Titratable Fixed-Ratio Combination of Insulin Glargine Plus Lixisenatide in Type 2 Diabetes Inadequately Controlled on Basal Insulin and Metformin: the LixiLan-L Randomized Trial. *Diabetes care*. 2016;39(11):1972-1980.
44. Rosenstock J, Hanefeld M, Shamanna P, *et al*. Beneficial effects of once-daily lixisenatide on overall and postprandial glycemic levels without significant excess of hypoglycemia in type 2 diabetes inadequately controlled on a sulfonylurea with or without metformin (GetGoal-S). *Journal of diabetes and its complications*. 2014;28(3):386-392.
45. Riddle MC, Forst T, Aronson R, *et al*. Adding once-daily lixisenatide for type 2 diabetes inadequately controlled with newly initiated and continuously titrated basal insulin glargine: a 24-week, randomized, placebo-controlled study (GetGoal-Duo 1). *Diabetes care*. 2013;36(9):2497-2503.
46. Meneilly GS, Roy-Duval C, Alawi H, *et al*. Lixisenatide Therapy in Older Patients With Type 2 Diabetes Inadequately Controlled on Their Current Antidiabetic Treatment: The GetGoal-O Randomized Trial*. Diabetes Care*. 2017;40(4):485-493.
47. Muskiet MHA, Tonneijck L, Huang Y, *et al*. Lixisenatide and renal outcomes in patients with type 2 diabetes and acute coronary syndrome: an exploratory analysis of the ELIXA randomised, placebo-controlled trial. *The lancet diabetes and endocrinology*. 2018;6(11):859-869.
48. Davies M, Pieber TR, Hartoft-Nielsen ML, *et al*. Effect of Oral Semaglutide Compared With Placebo and Subcutaneous Semaglutide on Glycemic Control in Patients With Type 2 Diabetes: a Randomized Clinical Trial. *JAMA.* 2017;318(15):1460-1470.
49. Zinman B, Aroda VR, Buse JB, *et al*. Efficacy, Safety, and Tolerability of Oral Semaglutide Versus Placebo Added to Insulin With or Without Metformin in Patients With Type 2 Diabetes: The PIONEER 8 Trial. *Diabetes Care*. 2019;42(12):2262-2271.
50. Zinman B, Bhosekar V, Busch R, *et al*. Semaglutide once weekly as add-on to SGLT-2 inhibitor therapy in type 2 diabetes (SUSTAIN 9): a randomised, placebo-controlled trial. *The lancet diabetes and endocrinology*. 2019;7(5)356-367.
51. Husain M, Birkenfeld AL, Donsmark M, *et al*. Oral semaglutide and cardiovascular outcomes in patients with type 2 diabetes. *New England Journal of Medicine*. 2019;381(9):841-851.
52. Mosenzon O, Blicher TM, Rosenlund S*, et al*. Efficacy and safety of oral semaglutide in patients with type 2 diabetes and moderate renal impairment (PIONEER 5): a placebo-controlled, randomised, phase 3a trial. *The lancet diabetes and endocrinology.* 2019;7(7):515-527.
53. Marso SP, Bain SC, Consoli A, *et al*. Semaglutide and Cardiovascular Outcomes in Patients with Type 2 Diabetes. *The New England journal of medicine*. 2016;375(19):1834-1844.
54. Gerstein HC, Colhoun HM, Dagenais GR, *et al*. Dulaglutide and renal outcomes in type 2 diabetes: an exploratory analysis of the REWIND randomised, placebo-controlled trial. *The Lancet*. 2019;394(10193):131-138.
55. Holman RR, Bethel MA, Mentz RJ, *et al*. Effects of once-weekly exenatide on cardiovascular outcomes in type 2 diabetes. *New England Journal of Medicine*. 2017;377(13):1228-1239.
56. DeFronzo RA, TriplittC, Qu Y, *et al*. Effects of exenatide plus rosiglitazone on beta-cell function and insulin sensitivity in subjects with type 2 diabetes on metformin. *Diabetes care*. 2010;33(5):951-957.
57. Guja C, Frías JP, Somogyi A, *et al*. Effect of exenatide QW or placebo, both added to titrated insulin glargine, in uncontrolled type 2 diabetes: the DURATION-7 randomized study. *Diabetes, obesity & metabolism*. 2018;20(7):1602-1614.
58. Reusch J, Stewart MW, Perkins CM, *et al*. Efficacy and safety of once-weekly glucagon-like peptide 1 receptor agonist albiglutide (HARMONY 1 trial): 52-week primary endpoint results from a randomized, double-blind, placebo-controlled trial in patients with type 2 diabetes mellitus not controlled on pioglitazone, with or without metformin. *Diabetes, obesity & metabolism*. 2014;16(12):1257-1264.
59. Hernandez AF, Green JB, Janmohamed S, *et al*. Albiglutide and cardiovascular outcomes in patients with type 2 diabetes and cardiovascular disease (Harmony Outcomes): a double-blind, randomised placebo-controlled trial. *The Lancet.* 2018;392(10157):1519-1529.
60. NCT01733758. [https://clinicaltrials.gov/ct2/show/NCT01733758.](https://clinicaltrials.gov/ct2/show/NCT00849017.)
61. NCT02964247. [https://clinicaltrials.gov/ct2/show/NCT02964247.](https://clinicaltrials.gov/ct2/show/NCT00849017.)
62. Davies MJ, Bain SC, Atkin SL, *et al*. Efficacy and Safety of Liraglutide Versus

Placebo as Add-on to Glucose-Lowering Therapy in Patients With Type 2 Diabetes and Moderate Renal Impairment (LIRA-RENAL): A Randomized Clinical Trial. *Diabetes care*. 2016;39(2):222-230.

1. Gough SC, Bode B, Woo V, *et al*. Efficacy and safety of a fixed-ratio combination of insulin degludec and liraglutide (IDegLira) compared with its components given alone: Results of a phase 3, open-label, randomised, 26-week, treat-to-target trial in insulin-naive patients with type 2 diabetes. *The Lancet Diabetes and Endocrinology*. 2014;2(11):885-893.
2. Buse JB, Vilsbøll T, Thurman J, *et al*. Contribution of liraglutide in the fixed-ratio combination of insulin degludec and liraglutide (IDegLira). *Diabetes care.* 2014;37(11):2926-2933.
3. Nauck M, Frid A, Hermansen K, *et al*. Efficacy and safety comparison of liraglutide, glimepiride, and placebo, all in combination with metformin, in type 2 diabetes: the LEAD (liraglutide effect and action in diabetes)-2 study. *Diabetes care.* 2009;32(1):84-90.
4. Pratley R, Amod A, Hoff ST, *et al*. Oral semaglutide versus subcutaneous liraglutide and placebo in type 2 diabetes (PIONEER 4): a randomised, double-blind, phase 3a trial. *Lancet*. 2019;394(10192):39-50.
5. Mann JFE, Ørsted DD, Brown-Frandsen K, *et al*. Liraglutide and Renal Outcomes in Type 2 Diabetes. *New England journal of medicine*. 2017;377(9): 839-848.
6. DeFronzo RA, Lee C, Kohler S. Safety and Tolerability of Combinations of Empagliflozin and Linagliptin in Patients with Type 2 Diabetes: pooled Data from Two Randomized Controlled Trials. *Advances in therapy*. 2018;35(7):1009-1022.
7. Rosenstock J, Hansen L, ZeeP, *et al*. Dual add-on therapy in type 2 diabetes poorly controlled with metformin monotherapy: a randomized double-blind trial of saxagliptin plus dapagliflozin addition versus single addition of saxagliptin or dapagliflozin to metformin. *Diabetes care*. 2015;38(3):376-383.
8. Lavalle-González FJ, Januszewicz A, Davidson J, *et al*. Efficacy and safety of canagliflozin compared with placebo and sitagliptin in patients with type 2 diabetes on background metformin monotherapy: a randomised trial. *Diabetologia*. 2013;56(12):2582-2592.
9. Pratley RE, Eldor R, Raji A, *et al*. Ertugliflozin plus sitagliptin versus either individual agent over 52 weeks in patients with type 2 diabetes mellitus inadequately controlled with metformin: the VERTIS FACTORIAL randomized trial. *Diabetes, obesity & metabolism*. 2018;20(5):1111-1120.
10. [Scott R](https://pubmed.th.dataotetao.com/pubmed/?term=Scott R[Author]&cauthor=true&cauthor_uid=30019498), [Morgan J](https://pubmed.th.dataotetao.com/pubmed/?term=Morgan J[Author]&cauthor=true&cauthor_uid=30019498), [Zimmer Z](https://pubmed.th.dataotetao.com/pubmed/?term=Zimmer Z[Author]&cauthor=true&cauthor_uid=30019498), [Lam RLH](https://pubmed.th.dataotetao.com/pubmed/?term=Lam RLH[Author]&cauthor=true&cauthor_uid=30019498), [O'Neill EA](https://pubmed.th.dataotetao.com/pubmed/?term=O'Neill EA[Author]&cauthor=true&cauthor_uid=30019498), [Kaufman KD](https://pubmed.th.dataotetao.com/pubmed/?term=Kaufman KD[Author]&cauthor=true&cauthor_uid=30019498), *et al*. A randomized clinical trial ofthe efficacy and safety of sitagliptin compared with dapagliflozin in patients with type 2 diabetes mellitus and mild renal insufficiency: The CompoSIT-R study. [*Diabetes Obes Metab*.](https://pubmed.th.dataotetao.com/pubmed/?term=A+randomized+clinical+trial+of+the+efficacy+and+safety+of+sitagliptin+compared+with+dapagliflozin+in+patients+with+type+2+diabetes+mellitus+and+mild+renal+insufficiency:+The+CompoSIT‐R+study) 2018;20(12):2876-2884.
11. Rosenstock J, Allison D, Birkenfeld AL, *et al*. Effect of Additional Oral Semaglutide vs Sitagliptin on Glycated Hemoglobin in Adults With Type 2 Diabetes Uncontrolled With Metformin Alone or With Sulfonylurea: the PIONEER 3 Randomized Clinical Trial. *JAMA*. 2019;321(15):1466-1480.
12. Ahrén B, Masmiquel L, Kumar H, *et al*. Efficacy and safety of once-weekly semaglutide versus once-daily sitagliptin as an add-on to metformin, thiazolidinediones, or both, in patients with type 2 diabetes (SUSTAIN 2): a 56-week, double-blind, phase 3a, randomised trial. *The lancet diabetes and endocrinology*. 2017;5(5):341-354.
13. Rodbard HW, Rosenstock J, Canani LH, *et al.* Oral Semaglutide versus Empagliflozin in Patients with Type 2 Diabetes Uncontrolled on Metformin: The PIONEER 2 Trial. *Diabetes care*.2019;42(12):2272-2281.
14. Pieber TR, Bode B, Mertens A, *et al*. Efficacy and safety of oral semaglutide with flexible dose adjustment versus sitagliptin in type 2 diabetes (PIONEER 7): a multicentre, open-label, randomised, phase 3a trial. *The lancet diabetes and endocrinology*. 2019;7(7):528-539.
15. Leiter LA, Carr MC, Stewart M, *et al*. Efficacy and safety of the once-weekly GLP-1 receptor agonist albiglutide versus sitagliptin in patients with type 2 diabetes and renal impairment: a randomized phase III study. *Diabetes care*. 2014;37(10):2723-2730.
16. Pratley R, Nauck M, Bailey T, *et al*. One year of liraglutide treatment offers sustained and more effective glycaemic control and weight reduction compared with sitagliptin, both in combination with metformin, in patients with type 2 diabetes: a randomised, parallel-group, open-label trial*. International journal of clinical practice.* 2011;65(4):397-407.
17. NCT00976937. [https://clinicaltrials.gov/ct2/show/NCT00976937.](https://clinicaltrials.gov/ct2/show/NCT00849017.)
